# Compensation by tumor suppressor genes during retinal development in mice and humans

**DOI:** 10.1186/1741-7007-4-14

**Published:** 2006-05-03

**Authors:** Stacy L Donovan, Brett Schweers, Rodrigo Martins, Dianna Johnson, Michael A Dyer

**Affiliations:** 1 Department of Developmental Neurobiology, St. Jude Children's Research Hospital, Memphis, TN 38105, USA; 2 Department of Ophthalmology, University of Tennessee Health Science Center, Memphis, TN 38163, USA

## Abstract

**Background:**

The *RB1 *gene was the first tumor suppressor gene cloned from humans by studying genetic lesions in families with retinoblastoma. Children who inherit one defective copy of the *RB1 *gene have an increased susceptibility to retinoblastoma. Several years after the identification of the human *RB1 *gene, a targeted deletion of *Rb *was generated in mice. Mice with one defective copy of the *Rb *gene do not develop retinoblastoma. In this manuscript, we explore the different roles of the Rb family in human and mouse retinal development in order to better understand the species-specific difference in retinoblastoma susceptibility.

**Results:**

We found that the Rb family of proteins (Rb, p107 and p130) are expressed in a dynamic manner during mouse retinal development. The primary Rb family member expressed in proliferating embryonic retinal progenitor cells in mice is p107, which is required for appropriate cell cycle exit during retinogenesis. The primary Rb family member expressed in proliferating postnatal retinal progenitor cells is Rb. p130 protein is expressed redundantly with Rb in postmitotic cells of the inner nuclear layer and the ganglion cell layer of the mouse retina. When Rb is inactivated in an acute or chronic manner during mouse retinal development, p107 is upregulated in a compensatory manner. Similarly, when p107 is inactivated in the mouse retina, Rb is upregulated. No changes in p130 expression were seen when p107, Rb or both were inactivated in the developing mouse retina. In the human retina, RB1 was the primary family member expressed throughout development. There was very little if any p107 expressed in the developing human retina. In contrast to the developing mouse retina, when *RB1 *was acutely inactivated in the developing human fetal retina, p107 was not upregulated in a compensatory manner.

**Conclusion:**

We propose that intrinsic genetic compensation between Rb and p107 prevents retinoblastoma in Rb- or p107-deficient mice, but this compensation does not occur in humans. Together, these data suggest a model that explains why humans are susceptible to retinoblastoma following *RB1 *loss, but mice require both *Rb *and *p107 *gene inactivation.

## Background

The timing of cell cycle exit is coordinated with cell-fate specification and differentiation in the developing central nervous system (CNS) to ensure that the appropriate number of neurons and glia are generated in the correct proportion. If too many neural progenitor cells exit the cell cycle during the early stages of development, then the overall number of cells is reduced, and the ratio of cell types shifts [1,2].

Genetically engineered mice carrying targeted deletions of tumor suppressor genes that regulate cell cycle progression have been used to study the coordination of proliferation and cell-fate specification during development. Mice lacking the retinoblastoma susceptibility gene, *Rb1 *[3-5], were among the first characterized. In embryonic day (E) 13.5 *Rb*^-/- ^mouse embryos, ectopic mitosis was observed outside the ventricular zone of the developing hindbrain, and concurrent apoptosis was observed in the region where postmitotic cells normally migrate and differentiate [4,5]. In contrast, the E13.5 *Rb*^-/- ^retina was indistinguishable from E13.5 wild-type or *Rb*-heterozygous retinas [3-6].

Lee and colleagues proposed that specific neuronal sublineages have unique requirements for Rb during development [5]. To explain the specific defects in neurogenesis in Rb-deficient animals, Jacks and colleagues suggested that other, closely related Rb proteins such as p107 play a more prominent role in certain lineages [4]. For example, tissues uniquely dependent on Rb (e.g., developing hindbrain) may exhibit defects in *Rb*^-/- ^embryos, but lineages that are not (e.g., embryonic retina) appear normal in its absence.

Although the Rb-deficient embryonic mouse retina develops normally up to E13.5, Rb may play an important role at later stages of development [7]. Using Cre-expressing retroviruses and a retinal progenitor-specific Cre transgenic mouse line (*Chx10-Cre*) mated to *Rb*^*Lox *^mice [8], we previously showed that the retinal defects first identified by Maandag and colleagues reflect a cell-autonomous requirement for Rb at specific developmental stages in retinal progenitor cells and rod photoreceptors [6,9]. Two other groups published similar findings using Cre transgenic mouse lines with broader expression in the retina [10] and throughout the CNS [11]. Other phenotypes characterized in *Rb*^-/- ^embryos were recently found to be secondary non-cell-autonomous effects caused by changes in extraembryonic tissues [12].

Analysis of mice carrying targeted deletions of *p107 *and *Rb *(*Rb*^+/-^; *p107*^-/-^) indicated that p107 is important for retinal development [13]. Mild retinal dysplasia was observed in the *Rb*^+/-^; *p107*^-/- ^mice at 4 to 6 months of age, and histologic analysis of postnatal day (P) 4 retinas suggested that dysplasia originated during development [13]. Subsequent studies using chimeric mice generated from *Rb*^-/-^; *p107*^-/- ^embryonic stem cells [14] and those involving conditional inactivation of *Rb *in the developing retina [10,15] or CNS [11] have not elucidated individual roles of Rb or p107 in retinogenesis.

When progenitor cell proliferation is disrupted during development, secondary effects on cell-fate specification and differentiation are likely to occur [1,2]. Therefore, distinguishing the roles of Rb and p107 in proliferation from those in cell-fate specification is a major challenge. In addition, elucidating the cell-autonomous and non-cell-autonomous [12] roles of the Rb family during development can be challenging [16]. Moreover, as several investigators have proposed [3-5,13,17], individual Rb proteins are likely to have both unique and overlapping roles during development, thereby making it difficult to identify their individual contributions. Compensation and redundancy can further complicate analyses and obscure the individual roles of Rb proteins during development [2,18,19]. Interestingly, the response to acute inactivation of *Rb *in mouse embryonic fibroblasts (MEFs) differs from that seen when *Rb *is inactivated in a chronic manner [20]. This finding increases the complexity of the genetic analyses of Rb family function in vivo.

Relatively little is known about the expression or the role of the Rb family during human fetal retinal development. Therefore, we do not know if Rb, p107 and p130 are playing different roles in the developing human and mouse retinas or if cell cycle regulation through the Rb family is evolutionarily conserved.

In this study, we used genetic tools (replication-incompetent retroviruses, in vivo electroporation, and SiRNA analyses) combined with cell cycle analysis to investigate the individual cell-autonomous roles of Rb and p107 in the developing retina. We also sought to compare Rb family expression and intrinsic genetic compensation during retinal development in mice and humans to explain why humans are susceptible to retinoblastoma following *RB1 *inactivation, but mice are not.

## Results

### Expression of the Rb family during mouse retinal development

**Figure 1:**
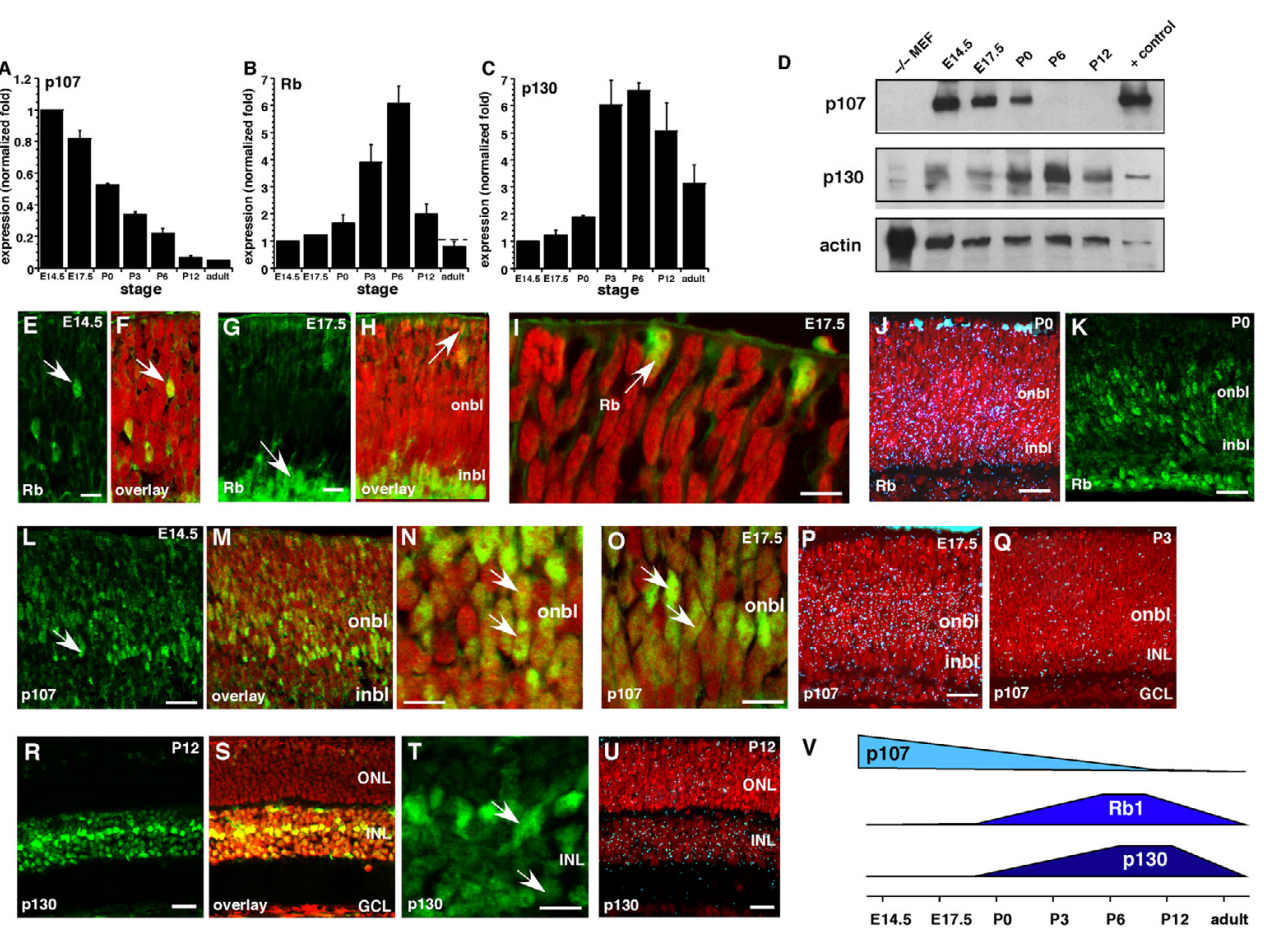
**Dynamic expression of the Rb family during mouse retinal development**. **(A-C) **Real-time PCR analysis of p107, Rb and p130 was done at seven stages of mouse retinal development. Data sets of three retinas per stage were analyzed twice, normalized to *Gapdh *and averaged. The standard deviations were within 5% of the mean. **(D) **Immunoblot analysis was done at five stages of mouse retinal development. For negative controls, MEF lysates prepared from knockout embryos were used. Positive controls were taken from 293T cells ectopically expressing the full-length cDNAs. Data were normalized to actin. **(E-I) **Immunofluorescent detection of Rb (green) in E14.5 and E17.5 retinas was overlaid on the nuclear counterstain (red). **(J) **Radioactive in situ hybridization analysis of Rb (blue silver grains) levels at P0 demonstrated broad expression in the onbl and inbl. This finding was consistent with immunofluorescent detection (green) of the protein **(K)**. **(L-O) **Immunofluorescent detection of p107 (green) in E14.5 and E17.5 retinas was overlaid on the nuclear counterstain (red). **(P) **Radioactive in situ hybridization for p107 (blue silver grains) at E17.5 demonstrated broad expression in the onbl and little expression at P3 **(Q)**. **(R-T) **Immunofluorescence of p130 at P12 (green) was overlaid on the nuclear counterstain (red). **(U) **Expression of p130 mRNA at P12 was consistent with the protein expression data. **(V) **Summary of the dynamic expression of the Rb family during retinal development. **Abbreviations: **GCL, ganglion cell layer; inbl, inner neuroblastic layer; INL, inner nuclear layer; MEF, mouse embryonic fibroblast; onbl, outer neuroblastic layer; ONL, outer nuclear layer. Scale bars: E, G, I, N, O and T, 10 μm; J, K, L, M, P, R and S, 25 μm.

Previous genetic studies have established that inactivation of Rb and p107 or Rb and p130 in the developing mouse retina can lead to retinoblastoma [6,10,11,14,15,21]. However, it is not known if Rb, p107 and p130 are expressed redundantly in the developing mouse retina or if intrinsic genetic compensation by *p107*, *p130 *or both prevents retinoblastoma in *Rb*-deficient mice. As a first step toward distinguishing between these two possibilities, we analyzed the expression of Rb, p107 and p130 over seven stages of mouse retinal histogenesis. Real-time RT-PCR and immunoblot analyses of E14.5 to P0 mice indicated that p107 is the primary Rb protein expressed in proliferating retinal progenitor cells (Fig. 1A, D). As the levels of p107 mRNA and protein decreased at P0, those of Rb and p130 increased (Fig. 1B–D) [6]. However, a low level of Rb was detected in a subset of embryonic retinal progenitor cells and newly postmitotic (transition) cells at the basal and apical surfaces of the developing inner neuroblastic layer (INBL) (Fig. 1E–K) [6].

The p107 mRNA and protein were restricted to proliferating retinal progenitor cells in the outer neuroblastic layer (ONBL) (Fig. 1L–Q), and p130 mRNA and protein were restricted to a subset of postnatal retinal progenitor cells, postmitotic neurons and glia in the inner nuclear layer (INL) and ganglion cell layer (GCL) during the final stages of retinal histogenesis (Fig. 1R–U and data not shown) [6]. These data indicated that the embryonic retinal progenitor cells primarily express p107 (Fig. 1V), which is consistent with a lack of any retinal phenotype in *Rb*^-/- ^embryonic retinas. In the postnatal retina and in differentiated neurons and glia of the INL and GCL, Rb and p130 were expressed redundantly (Fig. 1V). Rb is the only family member expressed in rod photoreceptors, a finding that is consistent with its unique role in the development of this neuronal cell type [6]. Together, these data suggest that Rb and p107 are expressed in a largely nonoverlapping pattern during mouse retinal development, which raises the possibility of compensation by p107 when Rb is inactivated. Moreover, Rb and p130 expression overlaps, which supports the idea that these proteins play redundant roles in a subset of retinal neurons.

### Expression of the Rb family during the cell cycle in retinal progenitor cells

**Figure 2:**
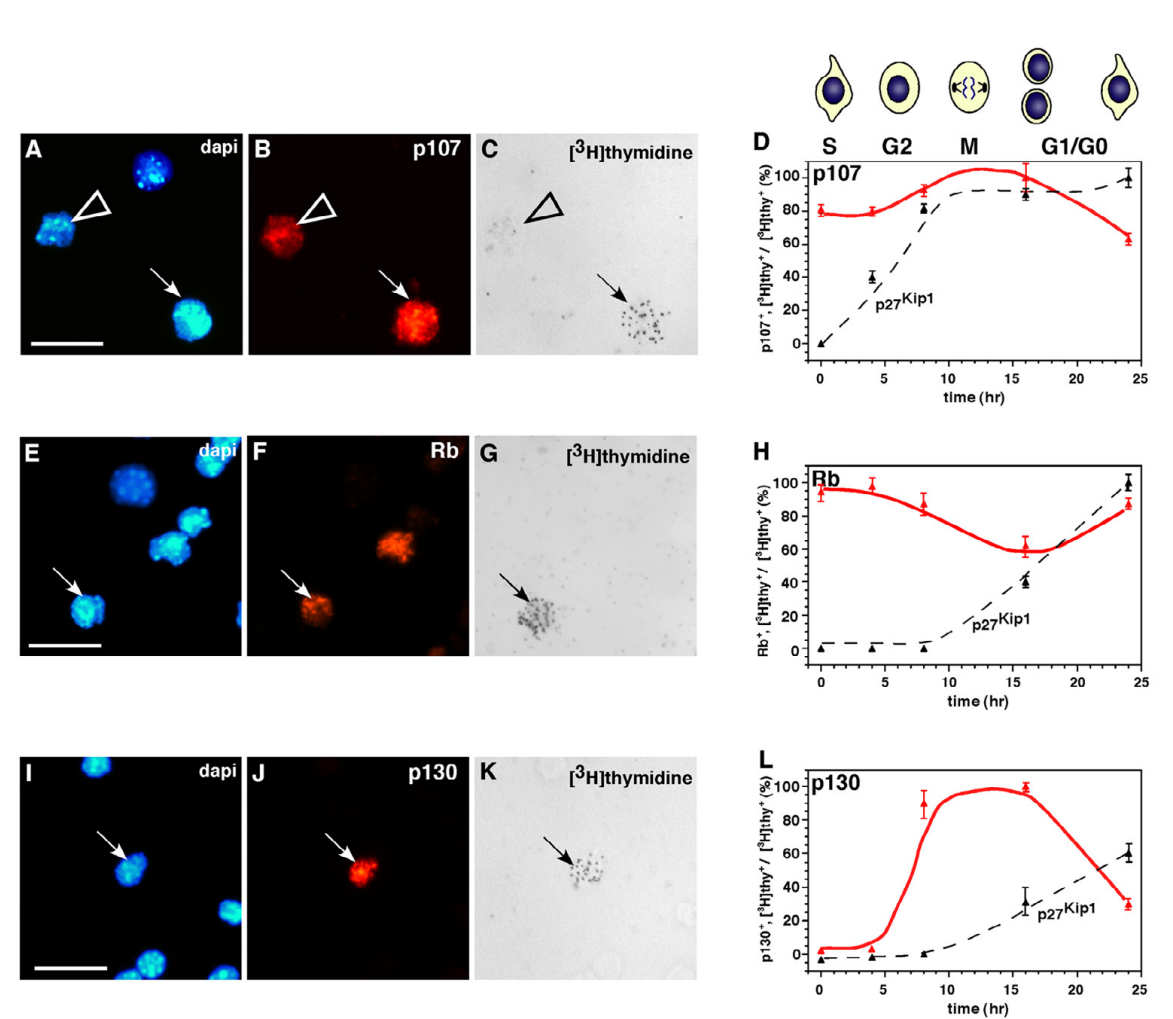
**Expression of the Rb family during the cell cycle**. **(A-D) **The E14.5 retinal explants were maintain in culture in the presence of [^3^H]thy for 1 h, washed and then maintained in culture for different periods. Dissociated retinas (dapi in **A**) were then immunostained for p107 (red in **B**) and overlaid with autoradiographic emulsion to detect the [^3^H]thy **(C)**. The proportion of p107+ cells was then scored (250 cells in duplicate), and normalized data were plotted **(D)**. The peak level of p107 was detected after 8 h of exposure, which coincided with the G2 and M phases of the cell cycle, and declined at 24 h, which coincided with the G1/G0 phase. **(E-H) **Similar experiments were done using P0 retinal explants and in P2 retinal explants **(I-L)**. **(H) **The proportion of [^3^H]thy-labeled Rb+ cells from P0 retinal explants was high during 0 to 4 h of exposure (S, G2) and subsequently declined. **(L) **The level of [^3^H]thy-labeled p130+ cells in P2 retinal explants was low during the first 4 h (S, G2), peaked at 8 and 16 h (M phase) and then declined during G1/G0. Similar data for p27 are plotted as an internal reference for cell cycle phase estimates. Scale bars: A, E and I, 10 μm.

Beyond determining when and where each Rb family member is expressed during retinal development, it is important to determine when during the cell cycle Rb, p107 and p130 are expressed. These data may help to elucidate their normal roles during development. For example, if a family member is only expressed in proliferating retinal progenitor cells, then that protein is unlikely to play a role in neuronal differentiation. It has been well established in a variety of culture systems that Rb, p107 and p130 are expressed during different phases of the cell cycle. To directly determine when during the cell cycle these proteins are expressed throughout retinal development, we scored the colocalization of [^3^H]thymidine ([^3^H]thy) with p107 (Fig. 2A–D), Rb (Fig. 2E–H) or p130 (Fig. 2I–L) at five time points during four stages of development [2,22,23]. The expression of p27^Kip1 ^has been characterized previously [22], and a parallel set of samples was analyzed for p27^Kip1 ^expression to serve as an internal control. The p27^Kip1 ^protein is upregulated as cells exit the cell cycle throughout retinal development, and the kinetics are different at E14.5, P0 and P2, because the length of the retinal progenitor cell cycle increases during retinogenesis [24]. At E14.5, 62% to 77% of proliferating cells expressed p107, and 4% to 6% expressed Rb (see Additional file 1). The 36% reduction in the proportion of p107+ cells at the 24-h time point in E14.5 retinas was consistent with the previous finding that 20% to 25% of rat retinal progenitor cells exit the cell cycle at a similar stage [22,24,25].

At P0, 76% to 87% of proliferating retinal progenitor cells expressed Rb (see Additional file 1; Fig. 2E–H), and the remaining cells expressed p107 (data not shown). Unlike p107+ cells, Rb+ P0 retinal progenitor cells continued to express Rb after they exited the cell cycle (compare Fig. 2H and 2D). In the P2 retina, the level of p130 peaked in the late G2/early M phase of the cell cycle and persisted into G1/G0 (see Additional file 1; Fig. 2I–L). Many cells downregulated p130 as they exited the cell cycle; this finding was consistent with the birth of rod photoreceptors (80% of the total cell population in the retina), which do not express p130 (Fig. 1R–U). Results from the [^3^H]thy pulse-labeling experiment combined with those from the immunostaining, real-time RT-PCR, in situ hybridization and immunoblot studies (Fig. 1) suggested that p107 is expressed primarily in retinal progenitor cells during embryonic retinal development; Rb is expressed in both retinal progenitor cells and postmitotic neurons and glia in the postnatal retina, and p130 is expressed in cells exiting the cell cycle during the late stages of retinal development. These data help us to further refine the possible compensatory and redundant roles of Rb, p107 and p130 during retinal development. Specifically, Rb and p130 may play redundant roles in postnatal retinal progenitor cells and postmitotic differentiated neurons of the INL and GCL. Rb and p107 are both expressed in proliferating retinal progenitor cells, albeit at different stages of development. Inactivation of either Rb or p107 in proliferating retinal progenitor cells may lead to compensatory upregulation of the other family member.

### Reciprocal compensation between Rb and p107 in mouse retinal progenitor cells

**Figure 3:**
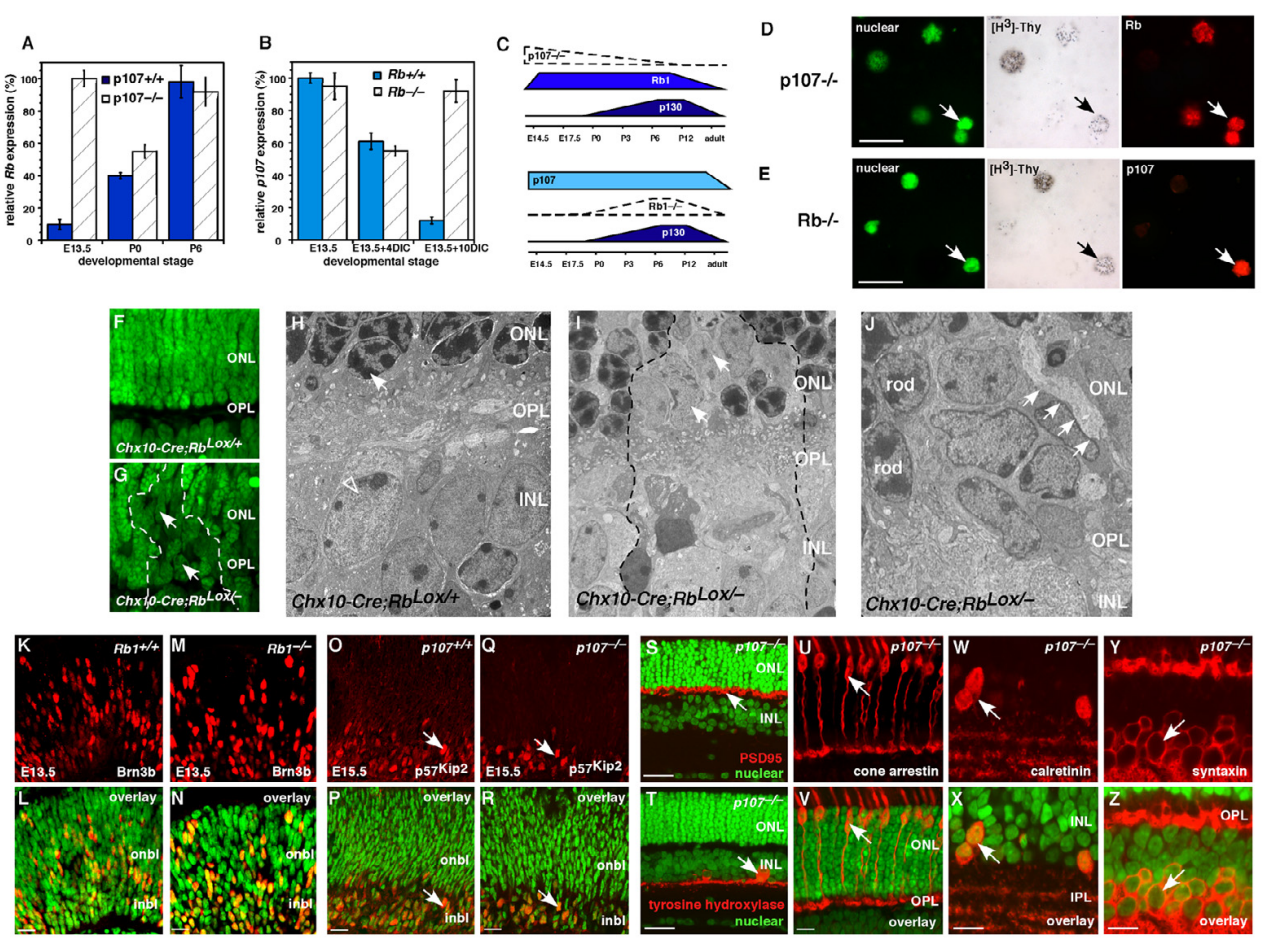
**Reciprocal compensation after *Rb *or *p107 *inactivation**. **(A) **Real-time PCR analysis of Rb expression in retinas from three developmental stages of p107-deficient or wild-type mice. At E13.5, Rb was upregulated. Analysis was done in triplicate samples analyzed in duplicate. **(B) **In a similar experiment, p107 expression was analyzed in Rb-deficient retinas. Due to the embryonic lethality of deleting *Rb*, cultured retinas were used for the later stages; p107 was upregulated in a compensatory manner. **(C) **A summary of the changes in Rb family gene expression after *Rb *or *p107 *inactivation. **(D) **E13.5 p107-deficient retinas were labeled with [^3^H]thy for 4 h and immunostained for Rb expression (red). **(E) **In a similar experiment, cultured Rb-deficient retinas were labeled for p107 expression. **(F, G) **In the absence of Rb in *Chx10-Cre;Rb*^*Lox*/- ^P12 retinas, rod photoreceptors failed to form, and chromatin failed to condense in the ONL (arrow). **(H) **Chromatin (arrow) in rod photoreceptors normally condenses during differentiation. **(I) **In the absence of Rb, rods remained immature with diffuse chromatin (arrow). **(J) **These defects in cell-fate specification also caused defects in synaptogenesis of horizontal cells (arrows); with processes extending apically into the ONL. **(K-N) **Wild-type and Rb-deficient E13.5 retinas were immunostained with an antibody against Brn3b (red), a ganglion cell marker. Brn3b was distributed across the retina (green nuclear stain overlay) and was indistinguishable in the presence or absence of Rb. **(O-R) **Wild-type and p107-deficient E15.5 retinas were immunostained for p57^Kip2 ^(red), which was expressed in a subset of retinal progenitor cells and newly postmitotic amacrine cells. **(S**) PSD95 (red) was expressed in the OPL and was normally distributed in the absence of p107. **(T) **Amacrine cells stained with tyrosine hydroxylase were also distributed normally. **(U, V) **Cone photoreceptors, **(W, X) **calretinin+ amacrine cells and **(Y, Z) **amacrine cells and horizontal cells were distributed normally in the *p107*-knockout retinas. **Abbreviations: **DIC, days in culture; INL, inner nuclear layer; IPL, inner plexiform layer; ONL, outer nuclear layer; OPL, outer plexiform layer. Scale bars: B, D, F, H, L, N, P, T and U, 10 μm; I and J, 25 μm.

To directly determine whether Rb family members compensate for each other in the developing retina, we isolated RNA from *p107*^-/-^, *p107*^+/-^, *p107*^+/+^, *Rb*^-/-^, *Rb*^+/- ^and *Rb*^+/+ ^retinas at E13.5, P0 and P6 for real-time PCR analysis. In E13.5 retinas lacking p107, Rb mRNA was upregulated in a compensatory manner (Fig. 3A). Similarly, in postnatal retinas lacking Rb, p107 mRNA was upregulated (Fig. 3B, C). The expression of p130 did not change significantly in any of the samples (data not shown).

Next, we tested whether the Rb and p107 proteins were also upregulated in a compensatory manner in proliferating retinal progenitor cells. Retinas were pulse-labeled with [^3^H]thy for 1 h, dissociated and immunostained for Rb and p107 (Fig. 3D, E). The mean proportion of Rb+ cells in the E13.5 *p107*^-/- ^retinas (54% ± 6%) was significantly higher than that in stage-matched wild-type retinas (4% ± 1.2%; *p *< 0.001). The mean proportion of p107+ cells in the P2 *Rb*^-/- ^retinas (7.3% ± 1.1%) was also significantly higher than that in age-matched wild-type retina (0.3% ± 0.2%; *p *< 0.005). The proportion of p107+ cells was lower than that of Rb+ cells, because there are fewer proliferating retinal progenitor cells at P2 than at E13.5 [24].

We have shown previously that Rb is required cell autonomously for the development of postmitotic rod photoreceptors [6,9]. To test whether p107 compensates for Rb in regulating the development of these postmitotic neurons, we performed electron microscopic (EM) analysis of *Chx10-Cre;Rb*^*Lox*/- ^retinas. One early morphologic feature of rod photoreceptor differentiation is chromatin condensation (Fig. 3F–H). In the absence of Rb, p107 was upregulated, but the cells fated to differentiate into rod photoreceptors, failed to condense their chromatin (Fig. 3I) and formed defective synapses in the outer plexiform layer (OPL) (Fig. 3J). These data suggest that the p107 compensation in the postnatal Rb-deficient mouse retina (Fig. 3B, C) helped to prevent deregulated proliferation but did not rescue the rod differentiation phenotype [6,9].

Rb is also expressed in a small subset of embryonic retinal progenitor cells (Fig. 1, 2; see Additional file 1), and subtle defects may occur in *Rb*^-/- ^embryonic retinas if p107 does not compensate at this stage of development. We immunostained *Rb*^-/-^, *Rb*^+/- ^and *Rb*^+/+ ^retinas at E13.5 with antibodies to several cell cycle proteins (p27^Kip1^, p57^Kip2^, cyclin D1, cyclin D3) and markers of differentiated cell types (Brn3b, GAP43, syntaxin, calbindin, cone arrestin). There was no difference among the three genotypes, in either the distribution or proportion of cells expressing any of these proliferation or differentiation markers (Fig. 3K–R and data not shown).

To determine if the compensation by Rb in p107-deficient retinas (Fig. 3A, C) prevented any defects in retinal progenitor cell proliferation, differentiation or both, we analyzed proliferation (BrdU labeling), apoptosis (TUNEL assay), cell-fate specification and neuronal differentiation (35 antibodies, see Methods) in *p107*^-/-^, *p107*^+/- ^and *p107*^+/+ ^retinas at E13.5, E15.5, P0, P3, P6 and P12. There was no significant difference among the genotypes at any stage examined (Fig. 3E–H). Even in vivo lineage studies using a replication-incompetent retrovirus in *p107*^-/-^, *p107*^+/- ^and *p107*^+/+ ^retinas revealed no defect in proliferation, survival, cell-fate specification or differentiation (Schweers and Dyer, in preparation). Together, these genetic analyses suggest that reciprocal compensation between Rb and p107 in retinal progenitor cells prevents deregulated proliferation during development. The p107 protein cannot compensate for Rb in postmitotic differentiating rods, and there was no evidence for p130 compensation in either Rb- or p107-deficient retinas at any stage of development.

### p107 compensation does not occur in the human fetal retina after *RB1 *inactivation

**Figure 4:**
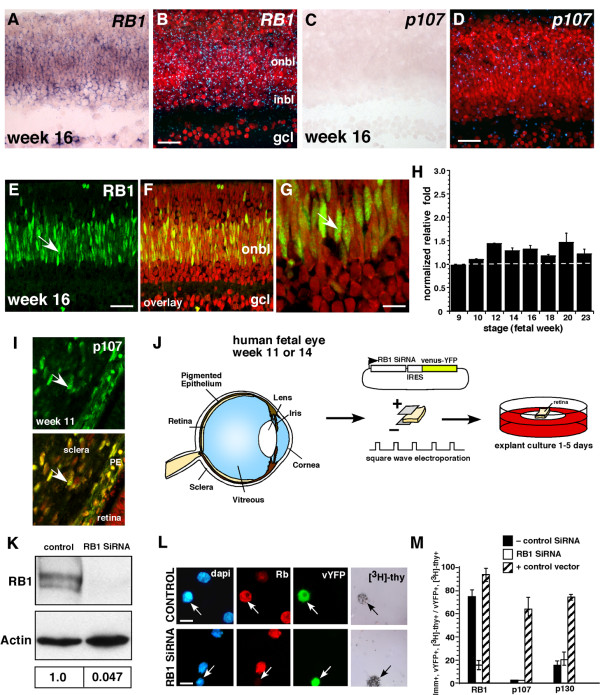
**Acute inactivation of *RB1 *in human fetal retinas**. **(A-D) **Representative in situ hybridization of fetal week 16 human retinas with DIG-labeled probes **(A, C) **and radioactively labeled probes **(B, D) **shows that *RB1 *is the major RB family member expressed in proliferating retinal progenitor cells during development. **(E-G) **Immunofluorescent detection of RB1 (green) in fetal week 16 human retinas confirmed that the protein is expressed in the same pattern as the mRNA. **(H) **Real-time RT-PCR analysis using TaqMan^® ^probes for *RB1 *demonstrated that the expression level does not dramatically change over the course of retinal histogenesis. Data sets were analyzed twice, normalized to *Gapdh *expression and averaged. **(I) **The sclera and pigmented epithelium were positive controls for p107 expression. **(J) **To inactivate *RB1 *in human fetal retinas, primary tissue was square-wave electroporated with a plasmid that encoded an siRNA to *RB1 *and a venus-YFP reporter gene. Retinas were then maintained in culture for several days and analyzed for compensation by p107. **(K) **COS cells transfected with the *RB1 *SiRNA vector shown in (**J**) showed a 21-fold reduction in the level of RB1 protein. Densitometry of normalized values for RB1 is shown in the lower portion of panel (**K**). **(L, M) **The venus-YFP+ retinal cells that were also [^3^H]thy+ downregulated RB1 but did not upregulate p107. The negative control SiRNA shown in (**M**) is the *Gapdh *SiRNA, but other nonspecific siRNAs gave similar results. The positive control samples are retinas that were square-wave electroporated with a plasmid expressing RB1, p107 or p130 and processed side-by-side with the SiRNA samples. **Abbreviations: **GCL, ganglion cell layer; inbl, inner neuroblastic layer; onbl, outer neuroblastic layer; PE, pigmented epithelium. Scale bars: G and L, 10 μm; B, D and E, 25 μm.

*RB1 *inactivation is sufficient for retinoblastoma initiation in humans, which suggests that p107 does not compensate or play a redundant role when *RB1 *is lost in human fetal retinas. To begin to address this question, we characterized the expression of the Rb family during human fetal retinal development by performing immunostaining, in situ hybridization and real-time RT-PCR analyses (Fig. 4A–H). Primary human fetal retinas from fetal weeks 9, 10, 11, 12, 14, 16, 18, 20 and 23 were analyzed; these stages of human fetal development correspond to the entire period of retinal progenitor cell proliferation (E14-P10) in the mouse [26]. RB1 is the primary family member expressed in proliferating retinal progenitor cells throughout human retinal development. Little p107 expression was detected by immunostaining, real-time RT-PCR or in situ hybridization analyses (Fig. 4C, D). An internal positive control for these expression studies was the surrounding sclera and pigmented epithelium that expressed high levels of p107 (Fig. 4I). During the late stages of retinal development, p130 was expressed in the retina in a pattern similar to that seen in mouse retinal development (data not shown).

To test if p107 compensation occurs when *RB1 *is inactivated, we performed square-wave electroporation to introduce the *RB1 *SiRNA and a venus-YFP reporter gene into human fetal week 11 retinas (Fig. 4J). The SiRNA knocked down RB1 protein expression by approximately 20 fold (Fig. 4K). An SiRNA to *Gapdh *and a scrambled *RB1 *SiRNA were used as negative controls. Retinas were maintained in culture for several days by using a procedure we optimized for mouse and primate fetal retinas (see Methods). Retinal progenitor cells proliferated normally with no appreciable cell death for at least 5 days while in this culture system (see Additional file 2). At several time points after electroporation, we labeled proliferating retinal progenitor cells with [^3^H]thy, dissociated the retinas, plated the cells on glass slides and immunostained them for RB1, p107 and p130 (Fig. 4L and data not shown). By scoring the expression of the Rb family in venus-YFP^+ ^cells that were also [^3^H]thy^+^, we found that RB1 was downregulated by the SiRNA 48 h after electroporation, but there was no compensation by p107 or p130 (Fig. 4M). An identical experiment carried out on fetal week 14 retinas gave similar results (data not shown). As positive controls for RB1, p107 and p130 immunostaining, we electroporated parallel cultures with a plasmid expressing these proteins (Fig. 4M). The expression data combined with that from the *RB1 *gene inactivation studies in primary human fetal retinas suggested that p107 is not normally expressed in the developing human retina, and it cannot compensate for *RB1 *loss in this tissue.

### Acute inactivation of Rb in the developing mouse retina

**Figure 5:**
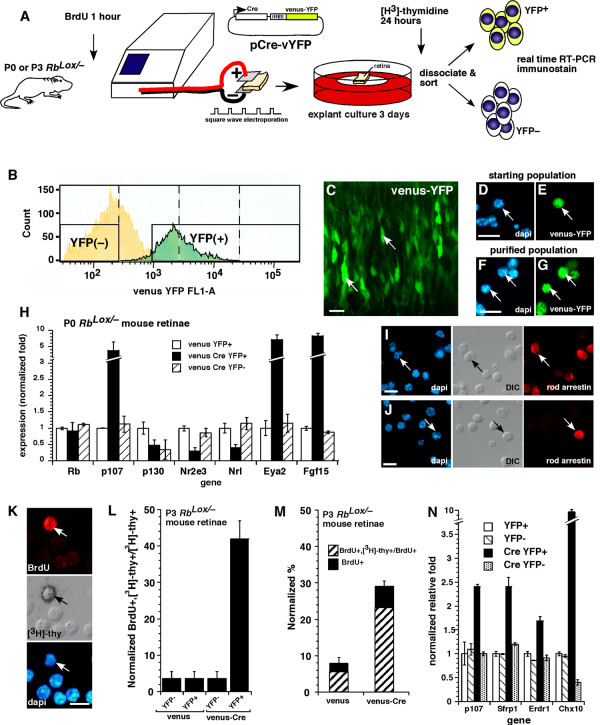
**Acute inactivation of *Rb *in the developing mouse retina**. **(A-G) **In vivo or in vitro square-wave electroporation and purification of cells with acute *Rb *inactivation. **(C) **A retina after electroporation is shown. **(D-G) **Dissociated cells before and after FACS purification are shown. **(H) **Real-time PCR analysis of YFP+ purified cells after acute *Rb *inactivation; each sample was analyzed in duplicate and normalized to *Gapdh *and *Gpi1*. **(I, J) **Immunostaining of purified cells after *Rb *inactivation. **(K-N) **To determine if retinal progenitor cells continue to divide after acute *Rb *inactivation, we scored the proportion of [^3^H]thy-labeled BrdU+ cells. An example of a double-positive cell is shown in **(K)**. **(L) **A significant proportion of progenitor cells was sensitive to deregulated proliferation after acute *Rb *inactivation, as indicated by the increase in double-positive cells. Data are normalized to account for the fraction of cells labeled with BrdU during a 1-h pulse. **(M) **The proportion of retinal progenitor cells that continue to divide after acute *Rb *inactivation is shown in the venus-Cre, YFP+ column. **(N) **Real-time PCR analysis of cell samples used in (**L) **and (**M) **revealed a significant increase in the expression of *p107 *and retinal progenitor cell markers *Sfrp1*, *Erdr1 *and *Chx10 *after acute *Rb *inactivation. Scale bars: C, D, F, I and J, 10 μm.

Compensation of p107 in MEFs depends on whether *Rb *is inactivated in an acute or chronic manner [20]. Our studies on *Rb*-knockout retinas including *Chx10-Cre;Rb*^*Lox*/- ^retinas showed that p107 was upregulated in a compensatory manner after chronic *Rb *inactivation in the retina. To determine whether this result occurs after acute *Rb *gene inactivation in the developing retina in vivo, we introduced a Cre-expressing plasmid (pCre-vYFP) into the eyes of newborn *Rb*^*Lox*/- ^mice (Fig. 5A). Real-time RT-PCR analysis of purified retinal cell populations (Fig. 5B–G) in which *Rb *was acutely inactivated showed that p107 mRNA was upregulated (Fig. 5H), as was seen after chronic *Rb *gene inactivation in the developing retina (Fig. 3). There was little change in *p130 *expression (Fig. 5H).

Next, we determined whether acute inactivation of *Rb *altered rod development. Real-time RT-PCR analysis of RNA samples indicated that the early photoreceptor genes *Nrl *and *Nr2e3 *were downregulated, as were several other photoreceptor-specific genes (Fig. 5H and data not shown). The cells that did not differentiate into photoreceptors resembled progenitor cells, as indicated by the increased expression of *Eya2 *and *Fgf15 *(Fig. 5H) [27]. To further verify these developmental defects, we immunostained the purified cells that had undergone acute *Rb *inactivation and compared them to the control cells (Fig. 5I, J). Those data were consistent with real-time PCR data showing that acute inactivation of *Rb *not only leads to a compensatory increase in *p107 *expression, but also disrupts rod development. Results of experiments in which *p107 *was acutely inactivated in wild-type E14.5 retinas by using an siRNA to p107 [20] were similar to those in the *p107*-knockout retinas, i.e., Rb was upregulated in a compensatory manner (data not shown).

Proliferating cells and nondividing or quiescent MEFs exhibit differences in the timing of their compensatory upregulation of p107 after *Rb *inactivation [20]. To test for differences in compensation in proliferating retinal progenitor cells and transition cells [28] in the developing retina, we acutely inactivated *Rb *at P3. Even though the majority (~ 95%) of cells in the P3 retina are postmitotic [24], a disproportionate number of proliferating retinal progenitor cells (42% ± 5.5%) underwent ectopic rounds of cell division (Fig. 5K, L; see Additional file 3). Retinal progenitor cells and transition cells were distinguished by BrdU labeling prior to electroporation (Fig. 5A). This finding suggests that retinal progenitor cells in which *Rb *has been acutely inactivated are susceptible to deregulated proliferation. A similar experiment in which all proliferating retinal progenitor cells in the P3 retina were labeled with [^3^H]thy for 24 h prior to acute *Rb *inactivation showed the same result (Fig. 5M). Specifically, even though retinal progenitor cells comprise only 7% of the total cell population at P3, they comprised 74% ± 4% of the cells that continued to divide after acute *Rb *inactivation (Fig. 5M). Real-time RT-PCR analysis of the samples demonstrated that *p107 *was upregulated in a compensatory manner and that retinal progenitor cell genes such as *Sfrp1*, *Erdr1 *and *Chx10 *were also upregulated (Fig. 5N).

To test if p107 compensation after acute *Rb *inactivation prevents deregulated proliferation, we acutely inactivated *p107 *by using a previously characterized SiRNA [20] in retinal cells in which *Rb *was chronically inactivated. We found that proliferation significantly increased from 18% ± 1.9% to 34% ± 1.9% (*p *= 0.013) after acute *p107 *inactivation (see Additional file 4). Moreover, the cells that continued to divide expressed retinal progenitor cell markers Chx10, Pax6 and syntaxin and were BrdU+ retinal progenitor cells at the time of *p107 *inactivation (see Additional file 4). Together, these data support the idea that p107 compensation is important for preventing deregulated proliferation in retinal progenitor cells whether *Rb *is inactivated in an acute or chronic manner.

### Haploinsufficiency of *p107 *in the absence of Rb

**Figure 6:**
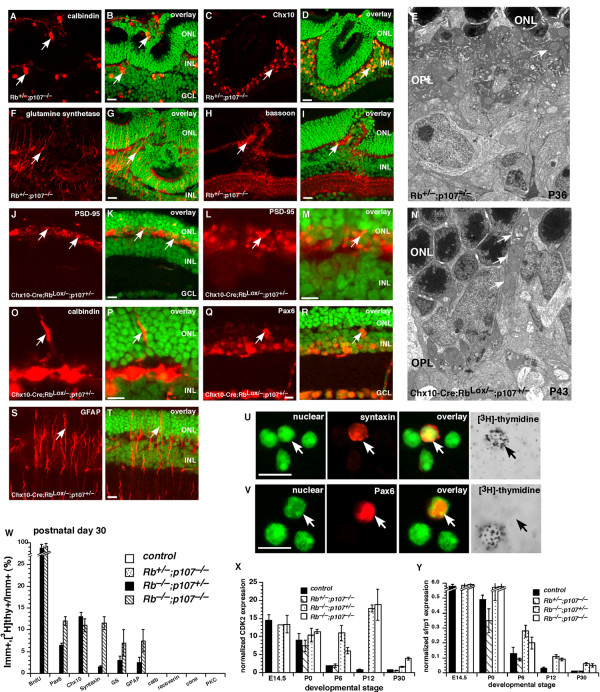
**Haploinsufficiency of *p107 *in the developing mouse retina**. Immunostaining of P30 *Rb*^+/-^; *p107*^-/- ^retinas in the region of the retinal dysplasia. **(A, B) **Antibodies against calbindin were used to identify horizontal cells; **(C, D) **those against Chx10, bipolar cells; **(F, G) **those against glutamine synthetase, Müller glia; and **(H, I) **those against bassoon, synapses in the OPL and IPL. **(E) **EM analysis of the P30 *Rb*^+/-^; *p107*^-/- ^retinas showing the OPL with normal organization of synaptic connections in contrast to the *Rb*-deficient retinas. **(J-T) **Immunostaining of P14 *Chx10-Cre;Rb*^*Lox*/-^; *p107*^+/- ^retinas in the region of Cre-mediated *Rb *inactivation using antibodies to OPL synapses (PSD-95, **J-M**), horizontal cells (calbindin, **O, P**), amacrine/progenitor cells (Pax6, **Q, R**), and reactive Müller glia (GFAP, **S, T**). **(N) **EM analysis of the P14 *Chx10-Cre;Rb*^*Lox*/-^; *p107*^+/- ^retinas showing the OPL with disrupted synaptic connections and an apical horizontal process (arrows). **(U-Y) **To determine if there are any ectopically dividing cells in *Rb*^+/-^; *p107*^-/-^, *Rb*^-/-^; *p107*^+/- ^or *Rb*^-/-^; *p107*^-/- ^retinas at E14.5, P0, P6, P12 and P30, we labeled retinas with [^3^H]thy and BrdU for 1 h and then dissociated and immunostained the cells with antibodies against 12 markers of different retinal cell types. BrdU and [^3^H]thy always colocalized. **(W) **The only markers that colocalized with [^3^H]thy were amacrine/progenitor cell markers Pax6 and syntaxin and bipolar/progenitor cell marker Chx10. There were also some reactive Müller glia, as indicated by GFAP, that incorporated [^3^H]thy. Ectopic proliferation was more prevalent in the double-knockout retinas than in the other genotypes. Each bar is the average of triplicate samples scored in duplicate. **(X, Y) **Real-time PCR analysis demonstrated that throughout development there were more progenitor cell markers such as *Cdk2 *and *Sfrp1 *expressed in the double-knockout and *Rb*^-/-^; *p107*^+/- ^retinas. **Abbreviations: **GCL, ganglion cell layer; INL, inner nuclear layer; ONL, outer nuclear layer; OPL, outer plexiform layer. Scale bars: B, D, G, I, K, M, P, Q, T, U and V, 10 μm.

Having shown that reciprocal compensation between Rb and p107 in the developing retina prevents deregulated proliferation of retinal progenitor cells, we tested whether a single copy of *Rb *was sufficient to compensate for p107 deficiency and whether a single copy of *p107 *was sufficient to compensate for Rb deficiency. Immunostaining of *Rb*^+/-^; *p107*^-/- ^retinas between P6 and P30 confirmed the presence of minor focal retinal dysplasia, as reported previously [13]. The laminar structure and synaptogenesis outside the regions of focal dysplasia were not substantially disrupted (Fig. 6A–I and data not shown), as confirmed by EM analysis of the OPL (Fig. 6E). In addition, EM analysis confirmed that rod photoreceptors [6] and horizontal cells [9] form normally with just one copy of *Rb *and no *p107 *(Fig. 6E). Even in the regions of the most severe dysplasia (Fig. 6A–I), cells were in their appropriate cellular layers, and synaptic markers were present in the corresponding plexiform layer sublaminae. EM analysis suggests that these focal dysplastic lesions are not formed by preneoplastic retinoblastoma cells. That is, there is no evidence for immature tumor cells localized to the lesions.

In contrast to the *Rb*^+/-^; *p107*^-/- ^retinas, the *Chx10-Cre;Rb*^*Lox*/-^; *p107*^+/- ^retinas exhibited much more severe developmental defects. Immunostaining revealed deregulated proliferation and lamination in an apical-basal mosaic pattern that was consistent with the expression of Cre from the *Chx10 *promoter of the *Chx10-Cre *transgene [9,29]. Rod photoreceptors were not completely lost due to the mosaic pattern of gene inactivation by the *Chx10-Cre *allele [29]. Defects in OPL synaptogenesis (Fig. 6J–T) were confirmed by EM analysis of P43 *Chx10-Cre;Rb*^*Lox*/-^; *p107*^+/- ^retinas (Fig. 6N). Müller glia showed upregulated expression of GFAP, which is a marker of reactive gliosis (Fig. 6S, T); however, GFAP expression was not detectable in Müller glia of the *Rb*^+/-^; *p107*^-/- ^retinas.

To quantify the proportion and types of cells that were proliferating in *Rb*^+/-^; *p107*^-/-^, *Rb*^-/-^; *p107*^+/- ^and *Rb*^-/-^; *p107*^-/- ^retinas during development, we labeled P0, P6, P12 and P30 retinas with [^3^H]thy for 1 h, dissociated the retinas and immunostained the cells with antibodies that recognized each of the retinal cell types (see Additional files 5,6,7,8). Few (if any) of the cells expressing differentiation markers such as recoverin (photoreceptors), cone arrestin (cones) or PKCα (bipolar cells) incorporated [^3^H]thy (Fig. 6U–W; see Additional files 5,6,7,8). Primarily, cells expressing progenitor cell markers (see Discussion) such as syntaxin, Pax6 or Chx10 incorporated [^3^H]thy (Fig. 6U–W; see Additional files 5,6,7,8). A small proportion of Müller glia also incorporated [^3^H]thy, a finding that was consistent with reactive gliosis-associated proliferation (Fig. 6S, T) [30]. The overall distribution of cell types was not significantly altered in *Rb*^+/-^; *p107*^-/- ^retinas (see Additional files 5,6,7,8), but as expected, the number of rods was reduced in *Chx10-Cre;Rb*^*Lox*/-^; *p107*^+/- ^retinas (see Additional files 5,6,7,8).

TUNEL assay results revealed no difference in the proportion of apoptotic cells in *Rb*^+/-^; *p107*^-/- ^retinas and that of their wild-type littermates. The number of apoptotic cells in *Chx10-Cre;Rb*^*Lox*/-^; *p107*^+/- ^retinas was slightly higher at P12 and P30, which corresponded to the stages of increased proliferation (see Additional files 5,6,7,8). Real-time RT-PCR analysis revealed increased expression of the retinal progenitor cell genes *Cdk2 *(Fig. 6X) and *Sfrp2 *(Fig. 6Y). Together, these data suggest that one copy of *Rb *in *Rb*^+/-^; *p107*^-/- ^retinas is sufficient for much of retinal development (with the exception of minor focal dysplasia), but one copy of *p107 *in *Chx10-Cre;Rb*^*Lox*/-^; *p107*^+/- ^retinas is haploinsufficient to regulate proliferation. Specifically, in retinal progenitor cells, two copies of the *p107 *gene are required to compensate for loss of *Rb*, but only one copy of *Rb *is required to compensate for loss of *p107*.

### Deregulated proliferation in Rb;p107-deficient retinal progenitor cells

**Figure 7:**
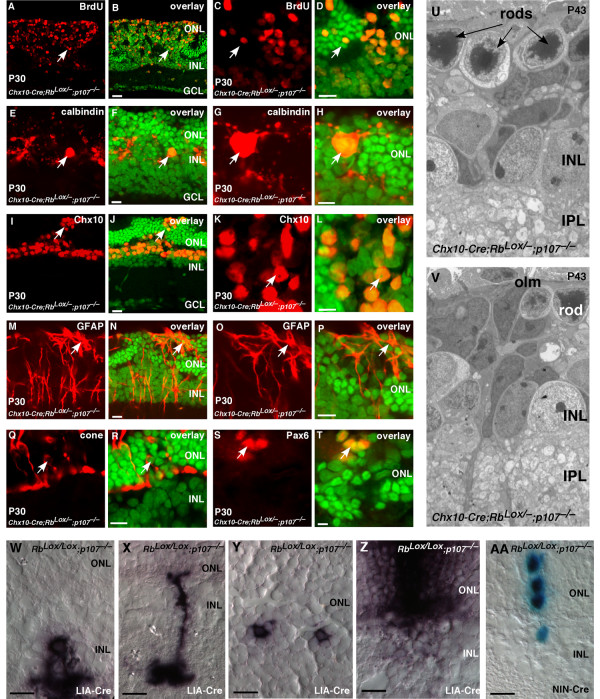
**Retinal progenitor cells continue to proliferate throughout development in the absence of Rb and p107**. **(A-T) **Immunostaining of P30 *Chx10-Cre;Rb*^*Lox*/-^; *p107*^-/- ^retinas by using antibodies against **(A-D) **BrdU to identify ectopically dividing cells; **(E-H) **calbindin, horizontal cells and Chx10, bipolar/progenitor cells; **(M-P) **GFAP, reactive Müller glia; **(Q, R) **cone arrestin, cone photoreceptors and **(S, T) **Pax6, amacrine/progenitor cells. **(U, V) **Electron micrographs showing that most retinal structures are disrupted in the absence of Rb and p107 and that continued proliferation eventually leads to retinoblastoma. **(W-Z) **Newborn *Rb*^*Lox*/*Lox*^; *p107*^-/- ^mice were injected with a retrovirus expressing Cre (LIA-Cre), and 3 weeks later, the retinas were sectioned and stained for alkaline phosphatase expression to identify the neurons that had lost Rb and p107. Amacrine cells (**W**) and bipolar cells (**X**) formed normally in the absence of Rb and p107. Rod photoreceptors (**Y**) failed to form normally. There were also several clones that spanned the INL or INL and ONL, which was indicative of hyperproliferation (**Z**). Similar experiments were carried out in E17.5 retinal explants by using a Cre-expressing retrovirus that also expresses nuclear LacZ (**AA**). These clones can readily be scored for cell number to determine if loss of Rb and p107 leads to deregulated proliferation. **Abbreviations: **GCL, ganglion cell layer; INL, inner nuclear layer; olm, outer limiting membrane; ONL, outer nuclear layer. Scale bars: B, D, F, H, J, L, N, P, R, T and W-AA, 10 μm.

It has been shown previously that retinoblastoma forms in *Rb;p107*-deficient retinas [10,11,14,15]. Therefore, we propose that reciprocal compensation between Rb and p107 in retinal progenitor cells prevents retinoblastoma in mice. If this is true, then we predict that retinal progenitor cells will continue to divide throughout development (E14-P30) in *Rb;p107*-deficient retinas. This is precisely what we found (Fig. 7A–D; see Additional files 5,6,7,8). The proliferating cells were detected in a mosaic pattern, which was consistent with *Rb *inactivation by the *Chx10-Cre *transgene (Fig. 7A–D) [9,29]. These immature proliferating cells disrupted synaptogenesis in the OPL (Fig. 7E–H) and expressed the progenitor cell markers syntaxin, Pax6 and Chx10 (Figs. 6W, 7I–L; see Additional files 5,6,7,8) [31-33]. With the exception of activated Müller glia (Fig. 7M–P; see Additional files 5,6,7,8), few of the proliferating cells expressed markers of differentiated retinal cells. It is important to point out that syntaxin, Pax6 and Chx10 are expressed in both retinal progenitor cells and postmitotic differentiated cells. However, the combination of BrdU, Pax6, Chx10 and syntaxin immunoreactivity combined with morphologic features of these cells in electron micrographs lends support to their identification as retinal progenitor cells.

In contrast to findings from a recent study [10], we did not detect any defect in cone photoreceptor production in retinal sections (Fig. 7Q, R) or in dissociated cells scored at P0, P6, P12 or P30 (see Additional files 5,6,7,8). EM analysis of *Chx10-Cre;Rb*^*Lox*/-^; *p107*^-/- ^retinas revealed immature cells spanning all cellular layers of the retina (Fig. 7U, V). These electron micrographs support the hypothesis that Chx10+, Pax6+, BrdU+ cells in the ONL and INL are retinal progenitor cells rather than differentiated cells. In addition, real-time PCR analysis demonstrated an increase in the expression of retinal progenitor cell markers in *Chx10-Cre;Rb*^*Lox*/-^; *p107*^-/- ^retinas at P6, P12 and P30 (Fig. 6X, Y).

Our analysis of the *Rb;p107*-deficient retinas over the course of postnatal retinal development showed that at all stages of development studied, there are ectopically dividing cells with morphologic features of retinal progenitor cells that express markers of retinal progenitor cells. Our data support the hypothesis that the role of reciprocal compensation between Rb and p107 is to prevent deregulated proliferation of retinal progenitor cells. If this is true, then we would expect that inactivation of *Rb *and *p107 *in a single proliferating retinal progenitor cell would lead to clonal expansion.

We infected proliferating retinal progenitor cells in newborn *Rb*^*Lox*/-^; *p107*^-/- ^mice with the Cre-expressing retrovirus LIA-Cre (Fig. 7W–Z). We observed significantly larger clone sizes and changes in cell-fate specification (Fig. 7Y, Z; see Additional file 9). To quantify the deregulated proliferation, we used the NIN-Cre retrovirus, which expresses nuclear lacZ and Cre, in E17.5 *Rb*^*Lox*/*Lox*^; *p107*^-/- ^retinas; NIN virus was used as a control. Clone size increased and was consistent with deregulated retinal progenitor cell proliferation. In control retinas infected with NIN, 248 of 313 (80%) clones were one cell, and 11 of 313 (3.5%) contained more than two cells. In retinas infected with NIN-Cre, 100 of 190 (52%) clones were one cell, and 42 of 190 (24%) contained more than two cells (Fig. 7AA). Similar results were seen in studies using the E1A 13S oncogene [6,15].

### Proliferation of progenitor cells and postmitotic transition cells in Rb;p107-deficient retinas

**Figure 8:**
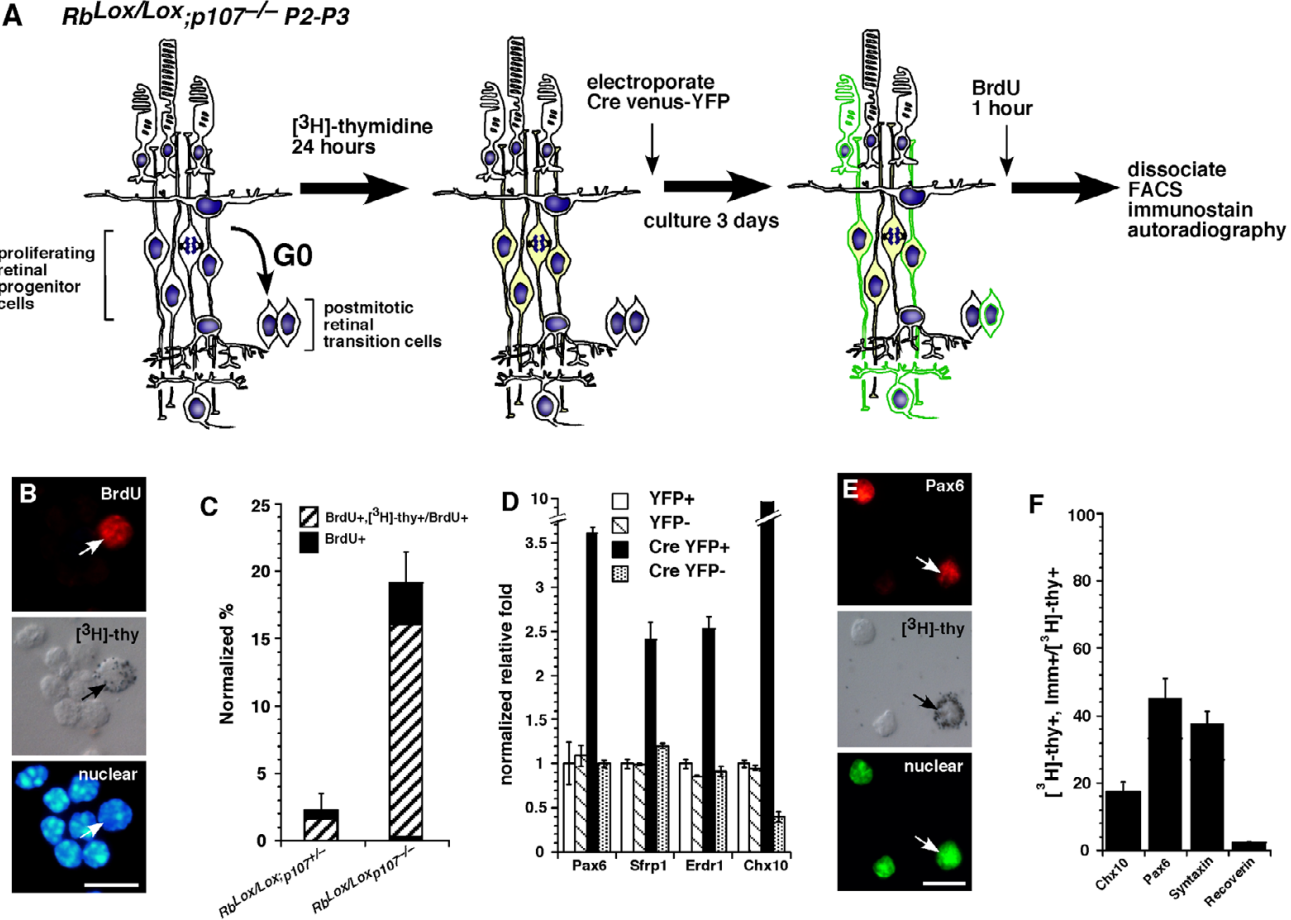
**Retinal progenitor cells are sensitive to deregulated proliferation after acute inactivation of *Rb *and *p107***. **(A) **To determine if retinal progenitor cells are sensitive to deregulated proliferation after acute *Rb *inactivation in a p107-deficient genetic background, we labeled *Rb*^*Lox*/*Lox*^; *p107*^-/- ^P0 or P3 retinas for 24 h with [^3^H]thy and then electroporated them with a Cre-venus-YFP plasmid. After 3 days in culture, the retinas were labeled for 1 h with BrdU and dissociated, and the YFP+ and YFP- cells were purified by FACS. **(B-F) **The cells were then analyzed by immunostaining, single-cell scoring and real-time PCR. **(B, C) **Colocalization of BrdU and [^3^H]thy demonstrated that most cells that continued to proliferate after *Rb *inactivation were retinal progenitor cells. **(D) **Real-time RT-PCR analysis confirmed that the proliferating cells expressed retinal progenitor cell markers. (**E, F**) Immunostaining and colocalization with [^3^H]thy further confirmed the retinal progenitor cell characteristics of these cells. Scale bars: B and E, 10 μm.

The developmental studies and clonal analyses described above suggest that reciprocal compensation between Rb and p107 prevents deregulated retinal progenitor cell proliferation and retinoblastoma. However, we have shown that p107 compensation also occurs at P3 when the majority (93%) of cells are postmitotic (Fig. 5N). Therefore, it is possible that reciprocal compensation between Rb and p107 prevents newly postmitotic cells from re-entering the cell cycle rather than preventing deregulated proliferation of retinal progenitor cells. To test this possibility directly, we labeled all proliferating retinal progenitor cells in P3 *Rb*^*Lox*/*Lox*^; *p107*^-/- ^retinas with [^3^H]thy (Fig. 8A), inactivated *Rb *expression by electroporating a plasmid that expressed Cre and vYFP and then incubated the retinas with BrdU. Cells that were BrdU+, vYFP+ and [^3^H]thy+ were retinal progenitor cells at the beginning of the experiment that continued to divide after acute *Rb *inactivation. In contrast, cells that were BrdU+, vYFP+ and [^3^H]thy- were postmitotic transition cells at the beginning of the experiment that re-entered the cell cycle after acute *Rb *inactivation. The time point P3 was selected because the vast majority (~ 93%) of cells in the retina at that stage are postmitotic [24]. If a postmitotic cell re-enters the cell cycle after acute *Rb *inactivation in *Rb*^*Lox*/*Lox*^; *p107*^-/- ^retinas, then the majority of BrdU+ cells would be [^3^H]thy-. If a retinal progenitor cell is more susceptible to deregulated proliferation after acute *Rb *inactivation, then we would predict that the majority of BrdU+ cells would be [^3^H]thy+. If both retinal progenitor cells and postmitotic cells divide after acute *Rb *inactivation, then the proportions of BrdU+, [^3^H]thy- cells and BrdU+, [^3^H]thy+ cells will reflect the proportions of postmitotic cells and mitotic cells in the starting population, respectively.

Most (79.8% ± 6%) ectopically dividing cells were BrdU+, [^3^H]thy+ cells (Fig. 8B, C). Consistent with this finding, results from real-time PCR and immunostaining analyses revealed that ectopically dividing cells expressed several retinal progenitor cell markers including *Pax6*, *Chx10*, *Syntaxin*, *Erdr1 *and *Sfrp1 *(Fig. 8D–F). However, there was a small increase in the proportion of BrdU+, [^3^H]thy- cells (Fig. 8C), which leaves open the possibility that either a retinal progenitor cell or a transition cell is the cell of origin for retinoblastoma (discussed in [28]).

## Discussion

We report here that in the developing mouse retina p107 is expressed in embryonic retinal progenitor cells, and Rb is expressed in postnatal retinal progenitor cells. When *p107 *is inactivated, Rb is upregulated in a compensatory manner, and when *Rb *is inactivated, p107 compensates. The p130 protein is expressed during the late stages of retinogenesis and persists in the INL and GCL in the adult retina. Rb and p130 are expressed redundantly in these cells. There was no evidence of compensatory changes in p130 expression following either *Rb *or *p107 *inactivation. Compensation by p107 occurred when *Rb *was inactivated acutely or chronically in the developing mouse retina. Two copies of the *p107 *gene were required to prevent deregulated proliferation of Rb-deficient retinal progenitor cells, making *p107 *haploinsufficient for compensation in the developing retina. In contrast, only one copy of *Rb *was required to regulate proliferation of p107-deficient retinal progenitor cells. We propose that reciprocal compensation between Rb and p107 prevents deregulated proliferation of retinal progenitor cells and retinoblastoma in mice. Similarly, redundant expression of Rb and p130 may help to prevent retinoblastoma in mice.

In contrast to mice, human fetal retinal progenitor cells primarily express RB1 during development. When *RB1 *was acutely inactivated in human fetal retinas, there was little if any compensatory upregulation of p107. This may explain why humans are susceptible to retinoblastoma following *RB1 *gene mutations, but mice require inactivation of *Rb *and *p107 *or *Rb *and *p130*.

### Overlapping and unique functions of the Rb family in the developing mouse retina

Immunostaining, in situ hybridization, real-time RT-PCR and immunoblot analyses revealed that p107 is expressed primarily in proliferating retinal progenitor cells of the embryonic mouse retina. Pulse-labeling with [^3^H]thy demonstrated that p107 is downregulated as cells exit the cell cycle. There were no defects in cell-fate specification or differentiation in p107-deficient retinas; thus, we propose that p107 primarily regulates retinal progenitor cell proliferation in the embryonic retina. Rb is expressed in postnatal retinal progenitor cells and differentiating neurons and glia. Consistent with a role in cell-fate specification or differentiation, Rb was expressed throughout the cell cycle and continued to be expressed as cells entered G0. In the absence of Rb, immature cells persist in the ONL and rod photoreceptors failed to form, a finding that indicates that Rb regulates the developmental processes in this specific sublineage.

Recent studies have extended these findings by focusing on the mechanism of Rb's role in rod development. Sun and colleagues generated an exon-specific knock-in allele of *Rb *with a single amino acid substitution (R654W) that reduces Rb binding to E2F1, E2F2 and E2F3 [34]. In *Chx10-Cre;Rb*^*654/Lox *^retinas, rods still fail to form. This finding led to a model in which Rb regulates a rod repressor gene through E2F1, E2F2 or E2F3, and in the absence of Rb, the rod lineage is blocked. Moreover, we predict that inactivation of the E2F that regulates this putative "rod repressor" would rescue the rod developmental defect. Preliminary data have shown that *Rb;E2F1*-deficient retinas have normal rod development, a finding that suggests that E2F1 is involved in this important developmental process.

Our data suggest that Rb and p107 share the same role in regulating mouse retinal progenitor cell proliferation but the role of Rb is unique in rods. An extensive genetic analysis of p130 is beyond the scope of this manuscript, but based on our expression studies and [^3^H]thy-labeling experiments, we predict that Rb and p130 are redundant in newly postmitotic cells of the INL and GCL. It is not known if p130 is required for terminal cell cycle exit in the developing retina, for maintaining cells in the postmitotic state or both.

A recent study published by Spencer and colleagues characterized the expression of the Rb family in developing mouse retina and adult human retina [35]. Our data differ from theirs, and we have presented the most relevant differences in Figure 1. Our analysis combined several independent methods (real-time RT-PCR, in situ hybridization, immunostaining of tissue sections, immunostaining of [^3^H]thy-labeled dissociated cells and immunoblotting); these approaches provided the same overall picture of the dynamic changes in the expression of the Rb family during seven stages of mouse retinal development. Spencer and colleagues provided a more limited data set with internal inconsistencies. For example, immunostaining was done on samples from just three stages of mouse retinal development (E14, P0 and P5), and in situ hybridization was carried out for Rb but not for p107 or p130. Their real-time RT-PCR and immunoblotting data show high levels of p107 expression in the embryonic retinas, but no p107+ nuclei are detected in their immunostained sections. Clearly, all expression data are limited by the reagents being used. For example, it is possible that the Rb, p107 and p130 expression patterns in our study or that of Spencer and colleagues are broader than reported due to the limited sensitivity of the antibodies used. We have attempted to minimize such limitations by combining several independent approaches such as in situ hybridization, immunostaining, real time RT-PCR and dissociated cell staining. Nontheless, we anticipate that the retinal expression pattern of the Rb family will be further refined as new reagents become available.

### Rb family redundancy and compensation in the developing mouse retina

The Rb family proteins are expressed in a dynamic manner during mouse retinal development. We show here that p107 is expressed in proliferating retinal progenitor cells in the embryonic retina and is downregulated as cells exit the cell cycle and undergo terminal differentiation. Around birth, the expression of p107 tapers off, and that of Rb takes it place in proliferating retinal progenitor cells. Rb expression persists in postmitotic differentiating neurons and glia of the mouse retina. The p130 protein is expressed in the late postnatal period of mouse retinal development. As cells exit the cell cycle, they upregulate p130, and its expression persists in postmitotic differentiating cells of the INL and GCL. Based on the expression pattern alone, one might predict that proliferation would be deregulated in p107-deficient embryonic retinas and Rb-deficient postnatal retinas. However, we report here that when p107 is inactivated, Rb is upregulated in a compensatory manner in the embryonic retina. Similarly, when Rb is inactivated, p107 is upregulated in a compensatory manner in the postnatal retina. There is no evidence for compensation by p130 in either Rb- or p107-deficient retinas.

One of the interesting features of the compensation that we uncovered in this study is the haploinsufficiency of *p107 *in this process. We show here that two copies of *p107 *are required for efficient compensation of *Rb *loss, but only one copy of *Rb *is required to compensate for *p107 *loss. This may indicate that there are different mechanisms for *Rb *and *p107 *gene compensation or that retinal progenitor cells exhibit different sensitivities to Rb and p107 protein levels. One intriguing possibility is that *p107 *haploinsufficiency for compensation may reflect the differential binding of Rb and p107 to different E2F family members.

It has been shown previously that *p107 *is an E2F-responsive gene; therefore, the mechanisms for p107 compensation most likely involve a direct feedback loop [36]. However, the mechanism for Rb compensation in the absence of p107 has not been elucidated. We propose that Rb-deficient retinal progenitor cells fail to form retinoblastoma because p107 is upregulated in a compensatory manner. However, it is possible that there is some overlapping, redundant expression around P0 in the developing mouse retina. Interestingly, Rb and p130 are expressed redundantly in the INL and GCL. The recent observation that retinoblastoma develops in *Rb;p130*-deficient retinas suggests that redundant expression of these two Rb family members prevents retinoblastoma in mice [11].

Previous studies using *Rb*^-/- ^MEFs demonstrated that inactivation of *Rb *in proliferating cells leads to compensatory upregulation of p107 and quiescence after serum starvation[20]. The *p107 *gene is persistently expressed in these quiescent *Rb*-deficient MEFs and is required for maintaining them in G0. When *Rb *was acutely inactivated in quiescent *Rb*^*Lox*/*Lox *^MEFs, p107 was not immediately upregulated in a compensatory manner, and the cells re-entered the cell cycle [20]. Therefore, chronic or acute inactivation of *Rb *can lead to very different outcomes in MEFs. The molecular changes that occur in MEFs as they become quiescent have been compared with those that accompany terminal cell cycle exit during development [20]. However, in the developing CNS, postmitotic neurons not only exit the cell cycle, but also migrate to their appropriate layer, extend axons and dendrites and form synaptic connections.

To test if there was any difference in p107 compensation following acute versus chronic *Rb *inactivation in the developing retina, we developed genetic tools [16] to acutely inactivate *Rb *in this context. Our data show that p107 compensation occurs when *Rb *is inactivated acutely or chronically in the developing retina. In addition, rod photoreceptors failed to form when *Rb *was acutely inactivated. These data suggest that p107 compensates for Rb in proliferating retinal progenitor cells, but it cannot take the place of Rb in differentiating rod photoreceptors.

We extended our acute inactivation studies to knock out *Rb *in *p107*-deficient cells and to knock out *p107 *in *Rb*-deficient cells. In these experiments, we compared the effects of acute gene inactivation on proliferating retinal progenitor cells and newly postmitotic transition cells. The newly postmitotic *Rb*-deficient transition cells in which *p107 *was acutely inactivated did not show extensive ectopic cell division. These data are consistent with the idea that p107 is important in regulating retinal progenitor cell proliferation but not in preventing newly postmitotic cells from re-entering the cell cycle. However, a small subset of postmitotic cells still re-entered the cell cycle following acute *Rb *inactivation in *p107-*deficient retinas. Therefore, we cannot rule out the possibility that the retinoblastoma cell of origin is a retinal transition cell [28]. Definitive proof will rely on following the expansion of clones lacking p107 and Rb to determine whether any rare transition cells re-enter the cell cycle and expand to generate retinoblastoma or if only retinal progenitor cells continue to proliferate, as shown here [28].

Our data on the proliferation of *Rb;p107*-deficient retinal cells during development contrasts with previous studies by Chen and colleagues [10]. We show that ectopically dividing retinal cells are present at P0, P6, P12 and P30, whereas the previous study argued that proliferation ceased at P30. This difference at P30 may be due to the more sensitive and reproducible [^3^H]thy labeling we used in our study compared to the BrdU labeling used by Chen et al. Alternatively, it could reflect the non-cell-autonomous effect of broad *Rb *inactivation in *Pax6-Cre;Rb*^*Lox*/*Lox*^; *p107*^-/- ^retinas examined by Chen et al. compared with the more restricted mosaic *Rb *inactivation in *Chx10-Cre;Rb*^*Lox*/-^; *p107*^-/- ^retinas that we investigated [16].

Another difference between our data and the study previously published by Chen and colleagues involves the interpretation of the markers expressed in proliferating cells of the *Rb;p107*-deficient retinas. We found that at P0, the ectopically dividing cells were largely Pax6+ (see Additional file 5). By P6, the proliferating cells were Pax6+, Chx10+ (see Additional file 6) and by P12, they were Pax6+, Chx10+ and syntaxin^+ ^(see Additional file 7). Pax6 [32] and syntaxin [31] are expressed in both retinal progenitor cells and differentiating amacrine cells. Chx10 is expressed in retinal progenitor cells and in differentiated bipolar cells and Müller glia [29]. Interestingly, at P12 we noted that GFAP+ Müller glia were also [^3^H]thy+. This finding is consistent with proliferation associated with reactive gliosis in *Rb;p107*-deficient retinas [30]. Chx10 immunopositivity may mark the proliferating Müller glia, bipolar cells or progenitor cells at this stage. However, we do not believe that the Chx10+ cells are bipolar cells, because there were no PKCα+ cells that incorporated [^3^H]thy at P12. At P30, most of the proliferating [^3^H]thy+ cells were syntaxin+. A smaller proportion were Pax6+, recoverin+ and PKCα+. Recoverin is expressed in a subset of bipolar cells as well as photoreceptors, so the PKCα+ and recoverin+ cells that incorporated [^3^H]thy are most likely a small cohort of bipolar cells that re-entered the cell cycle.

Based on the timeline of marker colocalization with [^3^H]thy that we present here, we propose that a Pax6+, Chx10+ and syntaxin+ retinal progenitor cell population that is biased toward the amacrine/horizontal cell fate continues to proliferate through development in *Chx10-Cre;Rb*^*Lox*/-^; *p107*^-/- ^retinas and eventually gives rise to retinoblastoma. This model is consistent with the extensive differentiation of mouse retinoblastomas along amacrine/horizontal cell lineage (Johnson and Dyer, in preparation). There is an occasional round of ectopic proliferation in reactive Müller glia and possibly bipolar cells at P30, but based on the absence of glial and bipolar markers in mouse retinoblastomas, we propose that these cells do not contribute significantly to retinoblastoma in mice.

Chen and colleagues [10] showed that Pax6+, Chx10+, Prox1+, Math3+, Crx+ and Hes5+ cells incorporated BrdU. With the possible exception of Crx, each of these markers is expressed in both progenitor cells and differentiated cells [18,32,33,37-42]. We have found that several additional retinal progenitor cell markers (syntaxin 1, Fgf15, Sfrp1, Erdr1 and Eya2) are expressed in proliferating cells of *Rb;p107*-deficient retinas. Overall, 10 of the 11 genes that are expressed in proliferating *Rb;p107*-deficient retinal cells have one feature in common; they are all expressed normally in retinal progenitor cells. The morphological features of the *Rb;p107*-deficient retinal cells in electron micrographs are also consistent with retinal progenitor cells. While these data support the hypothesis that retinal progenitor cells continue to divide during development of *Rb;p107*-deficient retinas and eventually give rise to retinoblastoma in mice, marker expression alone cannot distinguish between a progenitor cell and a transition cell as the cell of origin for retinoblastoma (discussed in [28]). Definitive proof of the cell of origin for retinoblastoma will require clonal analysis of tumor formation from a single cell in vivo.

### Retinoblastoma susceptibility in mice and humans

When the *Rb*-knockout mouse was generated over 13 years ago, it was expected that retinoblastoma would develop in *Rb*^+/- ^mice, as it does in *RB1*^+/- ^humans. However, that was not the case. We show here that the normal expression of Rb family members is dynamic during mouse retinal development and that reciprocal compensation between Rb and p107 may prevent retinoblastoma in mice.

In the human fetal retina, *RB1 *is the major family member expressed during much of retinal development. There is little or no expression of p107. We acutely inactivated *RB1 *in primary human fetal retinas by electroporating an SiRNA to *RB1*. We demonstrated that although the RB1 protein was downregulated 20-fold, there was no compensation by p107 or p130. These data suggest that humans are susceptible to retinoblastoma formation after *RB1 *inactivation, because they cannot upregulate p107 in a compensatory manner. It is possible that the 20-fold reduction in RB1 protein induced by the SiRNA used for these experiments is not sufficient to induce p107 compensation. However, we do not believe this is the case because another RB1 target gene (*p14*^*ARF*^) is induced by 10-fold following acute RB1 inactivation using the same SiRNA (Laurie et al., submitted). One possible explanation for the lack of compensation in the human retina is that the *p107 *promoter is sequestered in an inactive chromatin conformation in human fetal retina, but it is in an active chromatin conformation in mouse retinal progenitor cells and is thus poised to respond to a loss of Rb. Indeed, it is believed that promoters and genes that have been recently transcribed remain in an active chromatin configuration. Therefore, this model is consistent with the expression data for mouse and human *p107*. That is, the embryonic expression of p107 in the mouse retina results in an open chromatin conformation, thereby making it readily accessible for compensatory upregulation if *Rb *is inactivated. Detailed promoter and transcription analyses will be required to test this hypothesis.

## Conclusion

We have found that Rb and p107 are expressed in a largely non-overlapping pattern during mouse retinal development and that reciprocal compensation between these two Rb family members prevents retinoblastoma in mice. p107 compensation occurs whether Rb is inactivated chronically or acutely. In human retinal development, there is very little p107 expressed and compensation does not occur when RB1 is inactivated using an siRNA. We propose that this difference in compensation between mice and humans may explain why humans are susceptible to retinoblastoma following *RB1 *gene inactivation but mice require simultaneous inactivation of the *Rb *and *p107 *genes.

Our data also shed light on the retinoblastoma cell of origin. Proliferating retinal progenitor cells are disproportionately more susceptible to deregulated proliferation following *Rb;p107 *gene inactivation in comparison to newly postmitotic transition cells [28]. Consistent with this observation, 10 of 11 genes expressed in proliferating *Rb;p107*-deficient retinal cells are normally expressed in retinal progenitor cells. Electron micrographs of *Rb;p107*-deficient retinal cells provides further support for an immature cell with features of progenitor cells as the retinoblastoma cell of origin. However, based on our data, it remains a formal possibility that a postmitotic amacrine or horizontal cell is the retinoblastoma cell of origin. Ongoing lineage studies should help to determine if a progenitor cell, a postmitotic neuron or both can give rise to retinoblastoma in mice.

## Methods

### Mouse strains

*Rb*^+/- ^mice were obtained from The Jackson Laboratory (Bar Harbor, ME), and *Rb*^*Lox*/*Lox *^mice were obtained from the National Cancer Institute. The *p107*-knockout mice were obtained from Dr. Tyler Jacks (MIT). *Chx10-Cre *mice were obtained from Dr. Connie Cepko (Harvard Medical School). All mice were crossed to C57Bl/6 mice purchased from Charles River Laboratories (Wilmington, MA). The St. Jude Children's Research Hospital Institutional Animal Care and Use Committee approved all of the animal experiments.

### Antibodies, immunostaining, BrdU, [^3^H]thymidine and TUNEL studies

We immunolabeled retinal cryosections from mice of various genotypes (*p107*^-/-^, *p107*^+/-^, *p107*^+/+^) at various postnatal stages (P3, P6, P12 and adult) and dissociated retinas (500 cells per sample in triplicate) as previously described [22,43]. The list of antibodies used is provided (Supplementary Table 9). To label S-phase retinal progenitor cells, we incubated freshly dissected retinas in 1 ml explant culture medium containing [^3^H]thy (5 μCi/ml; 89 μCi/mmol) or 10 μM BrdU for 1 h at 37°C. For these pulse-labeling experiments, retinas were dissected at four stages of development (E14.5, E17.5, P0, and P2) and exposed to the label for various durations (0, 4, 8, 16 and 24 h) to allow retinal progenitor cells to progress through the cell cycle. The p27^Kip1 ^protein, which is upregulated at G1/G0, was used as an internal control (Fig. 2D, H, L), because its expression during retinal development has been characterized previously [2,22,23].

Autoradiography and BrdU detection were carried out as described previously [22,43]. For apoptosis analysis, we sectioned (14-μm) retinas on a cryostat. We used the colorimetric TUNEL apoptosis system (Promega, Madison, WI) per the manufacturer's instructions; however, for detection, we used tyramide-Cy3 (Perkin-Elmer, Wellesley, MA) rather than the colorimetric substrate.

### Electroporation and FACS

Retinas were electroporated in vivo at P0 by injecting 0.5 μl CsCl preparation plasmid DNA (5 μg/μL) into the eye. Electroporation consisted of five 50-μsec pulses of 80 V each separated by 950-μsec recovery periods. As a control, retinas were electroporated with a plasmid lacking Cre. For explant cultures, the DNA was purified and resuspended in HBSS at 1 μg/μL.

Electroporation consisted of five pulses of 25 V for 50 μsec each with 950-μsec recovery periods. For FACS purification of electroporated cells, retinas were dissociated as described previously [22,43], resuspended in explant culture medium and sorted using vYFP fluorescence on a Becton-Dickson FACS system (Rockville, MD).

### Real-time RT-PCR

Real-time RT-PCR experiments were performed using the ABI 7900 HT Sequence Detection System (Applied Biosystems, Foster City, CA). Primers and probes were designed using Primer Express^® ^software (Applied Biosystems). TaqMan^® ^probes were synthesized with 5'-FAM and 3'-BHQ. RNA was prepared using Trizol, and cDNA was synthesized using the Superscript system (Invitrogen, Carlsbad, CA). Samples were analyzed in duplicate and normalized to *Gapdh, Gpi1 *and *Mmt2 *expression levels.

### In situ hybridization and immunoblot analyses

For immunoblot analysis, retinas of C57Bl/6 mice were dissected at the following seven stages of development: E14.5, E17.5, P0, P3, P6, P12 and adult. Retinas were sonicated in RIPA with protease and phosphatase inhibitors (Sigma, St. Louis, MO) to clear the lysate. The debris was pelleted, and the concentration of each lysate was determined using a Bradford assay (BioRad Laboratories, Hercules, CA). Lysate (30 μg) was loaded onto each lane and transferred to nitrocellulose. Each antibody was used at a dilution of 1:1000, and HRP-conjugated secondary antibodies were used at 1:5000. The Amersham ECL system (Piscataway, NJ) was used to detect antibody binding per the manufacturer's instructions. For in situ hybridization, retinas from each stage of development were rapidly dissected, fixed for 1 h in 4% paraformaldehyde, cryoprotected in 30% sucrose/PBS and embedded in OCT before freezing on dry ice. Cryosections (14-μm thick) were cut, mounted on slides and dried. Tissue was rehydrated in and treated briefly with proteinase K. Slides were then acetylated in acetic anhydride/0.1 M TEA and dehydrated using an alcohol series. Prehybridization was carried out with formamide/SDS hybridization solution, and the riboprobes were added (^33^P-labeled or DIG-labeled probes at the same specific activity and total mass). Hybridization was carried out at 60°C overnight, and the slides were washed in a series of SSC washes. The sections were then counterstained with a nuclear dye (Sytox green or PI) and dehydrated prior to immersion in autoradiographic emulsion. Exposure was carried out for 1 week before the blots were developed.

### Human fetal retinal cultures

Human fetal eyes were provided by ABR, Inc. (Alameda, CA). Eyes were transported in RPMI medium on wet ice. Retinas were isolated and divided into 8 radial sections. Each section was placed on a polycarbonate filter and maintained in explant culture medium, as described previously for mouse retinas [22,25,37]. BrdU labeling, [^3^H]thy labeling, dissociation and FACS were carried out as described above for mouse retinas. These experiments were reviewed and approved by the St. Jude Children's Research Hospital Institutional Review Board.

### Microscopy

Bright-field and single-cell fluorescent images were obtained using a Zeiss Axioplan-2 fluorescent microscope with the Zeiss AxioCam digital camera. Fluorescent images of tissue sections were obtained using a Leica TCSNT confocal microscope. For EM, animals were anesthetized with avertin until a loss of deep tendon reflexes. Transcardial perfusion was performed with carboxygenated Ames Medium supplemented with 40 mM glucose to clear the vasculature, followed by perfusion with Sorenson's phosphate buffer (pH 7.2) containing 2% EM-grade paraformaldehyde and 1% EM-grade glutaraldehyde. Eyes were then harvested; a slit was made in the cornea to aid in diffusion; and the tissue was placed in 3% glutaraldehyde in Sorenson's phosphate buffer overnight. Tissue was washed with 0.2 M cacodylate buffer in 5% sucrose, post-fixed in 1% OsO_4_, embedded, sectioned and viewed by transmission EM.

### Retroviruses and retinal cultures

Retroviruses and retinal culture procedures have been extensively described elsewhere [22,37,43].

## Authors' contributions

SD carried out in situ hybridization for mouse and human Rb family genes and performed the square wave electroporation and FACS experiments on human and mouse retinae. BS analyzed the mouse retinae from the different genetic combinations of Rb and p107 deficient mice and performed the reciprocal compensation experiments. BS also assisted with single cell immunostaining and scoring and performed the real time RT-PCR these samples. RM developed and characterized the RB1 siRNA and performed the immunoblot experiments. DJ performed all EM analysis. MD performed the immunostaining of the Rb family in human and mouse retinae and performed the [^3^H]-thymidine pulse-labeling experiment. All authors contributed to the preparation of the manuscript.

## Supplementary Material

Additional file 1 Expression of the Rb, p107 and p130 during the cell cycle in retinal progenitor cells. Click here for file

Additional file 2 Human fetal retinal explant cultures. Click here for file

Additional file 3 Effects of acute inactivation of *Rb* in the developing retina. Click here for file

Additional file 4 Acute inactivation of *p107* in the *Rb*-deficient retinae during development. Click here for file

Additional file 5 Expression of Proliferation and Differentiation Markers in P0 Retinae Lacking Rb and/or p107. Click here for file

Additional file 6 Expression of Proliferation and Differentiation Markers in P6 Retinae Lacking Rb and/or p107. Click here for file

Additional file 7 Expression of Proliferation and Differentiation Markers in P12 Retinae Lacking Rb and/or p107. Click here for file

Additional file 8 Expression of Proliferation and Differentiation Markers in P30 Retinae Lacking Rb and/or p107. Click here for file

Additional file 9 Clone composition and distribution from in vivo lineage analysis. Click here for file

Additional file 10 Antibodies used for immunofluorescence on adult tissue. Click here for file

## References

[B1] Donovan SL, Dyer MA (2005). Regulation of proliferation during central nervous system development. Semin Cell Dev Biol.

[B2] Dyer MA, Cepko CL (2001). Regulating proliferation during retinal development. Nat Rev Neurosci.

[B3] Clarke AR, Maandag ER, van Roon M, van der Lugt NM, van der Valk M, Hooper ML, Berns A, te Riele H (1992). Requirement for a functional Rb-1 gene in murine development. Nature.

[B4] Jacks T, Fazeli A, Schmitt EM, Bronson RT, Goodell MA, Weinberg RA (1992). Effects of an Rb mutation in the mouse. Nature.

[B5] Lee EY, Chang CY, Hu N, Wang YC, Lai CC, Herrup K, Lee WH, Bradley A (1992). Mice deficient for Rb are nonviable and show defects in neurogenesis and haematopoiesis. Nature.

[B6] Zhang J, Gray J, Wu L, Leone G, Rowan S, Cepko CL, Zhu X, Craft CM, Dyer MA (2004). Rb regulates proliferation and rod photoreceptor development in the mouse retina. Nat Genet.

[B7] Maandag EC, van der Valk M, Vlaar M, Feltkamp C, O'Brien J, van Roon M, van der Lugt N, Berns A, te Riele H (1994). Developmental rescue of an embryonic-lethal mutation in the retinoblastoma gene in chimeric mice. Embo J.

[B8] Marino S, Vooijs M, van Der Gulden H, Jonkers J, Berns A (2000). Induction of medulloblastomas in p53-null mutant mice by somatic inactivation of Rb in the external granular layer cells of the cerebellum. Genes Dev.

[B9] Donovan SL, Dyer MA (2004). Developmental defects in Rb-deficient retinae. Vision Res.

[B10] Chen D, Livne-Bar I, Vanderluit JL, Slack RS, Agochiya M, Bremner R (2004). Cell-specific effects of RB or RB/p107 loss on retinal development implicate an intrinsically death-resistant cell-of-origin in retinoblastoma. Cancer Cell.

[B11] MacPherson D, Sage J, Kim T, Ho D, McLaughlin ME, Jacks T (2004). Cell type-specific effects of Rb deletion in the murine retina. Genes Dev.

[B12] Wu L, de Bruin A, Saavedra HI, Starovic M, Trimboli A, Yang Y, Opavska J, Wilson P, Thompson JC, Ostrowski MC (2003). Extra-embryonic function of Rb is essential for embryonic development and viability. Nature.

[B13] Lee MH, Williams BO, Mulligan G, Mukai S, Bronson RT, Dyson N, Harlow E, Jacks T (1996). Targeted disruption of p107: functional overlap between p107 and Rb. Genes Dev.

[B14] Robanus-Maandag E, Dekker M, van der Valk M, Carrozza ML, Jeanny JC, Dannenberg JH, Berns A, te Riele H (1998). p107 is a suppressor of retinoblastoma development in pRb-deficient mice. Genes Dev.

[B15] Zhang J, Schweers B, Dyer MA (2004). The First Knockout Mouse Model of Retinoblastoma. Cell Cycle.

[B16] Schweers B, Dyer MA (2005). New Genetic Tools for Studying Retinal Development and Disease. Vis Neurosci.

[B17] Cobrinik D, Lee MH, Hannon G, Mulligan G, Bronson RT, Dyson N, Harlow E, Beach D, Weinberg RA, Jacks T (1996). Shared role of the pRB-related p130 and p107 proteins in limb development. Genes Dev.

[B18] Dyer MA (2003). Regulation of proliferation, cell fate specification and differentiation by the homeodomain proteins Prox1, Six3, and Chx10 in the developing retina. Cell Cycle.

[B19] Dyer MA (2004). Mouse models of childhood cancer of the nervous system. J Clin Pathol.

[B20] Sage J, Miller AL, Perez-Mancera PA, Wysocki JM, Jacks T (2003). Acute mutation of retinoblastoma gene function is sufficient for cell cycle re-entry. Nature.

[B21] Dannenberg JH, Schuijff L, Dekker M, van der Valk M, te Riele H (2004). Tissue-specific tumor suppressor activity of retinoblastoma gene homologs p107 and p130. Genes Dev.

[B22] Dyer MA, Cepko CL (2001). p27Kip1 and p57Kip2 regulate proliferation in distinct retinal progenitor cell populations. J Neurosci.

[B23] Levine EM, Close J, Fero M, Ostrovsky A, Reh TA (2000). p27(Kip1) regulates cell cycle withdrawal of late multipotent progenitor cells in the mammalian retina. Dev Biol.

[B24] Alexiades MR, Cepko C (1996). Quantitative analysis of proliferation and cell cycle length during development of the rat retina. Dev Dyn.

[B25] Dyer MA, Cepko CL (2000). p57(Kip2) regulates progenitor cell proliferation and amacrine interneuron development in the mouse retina. Development.

[B26] Clancy B, Darlington RB, Finlay BL (2001). Translating developmental time across mammalian species. Neuroscience.

[B27] Blackshaw S, Harpavat S, Trimarchi J, Cai L, Huang H, Kuo WP, Weber G, Lee K, Fraioli RE, Cho SH, Yung R, Asch E, Ohno-Machado L, Wong WH, Cepko CL (2004). Genomic Analysis of Mouse Retinal Development. PLoS Biol.

[B28] Dyer MA, Bremner R (2005). The Search For the Retinoblastoma Cell of Origin. Nature Reviews Cancer.

[B29] Rowan S, Cepko CL (2004). Genetic analysis of the homeodomain transcription factor Chx10 in the retina using a novel multifunctional BAC transgenic mouse reporter. Dev Biol.

[B30] Dyer MA, Cepko CL (2000). Control of Muller glial cell proliferation and activation following retinal injury. Nat Neurosci.

[B31] Alexiades MR, Cepko CL (1997). Subsets of retinal progenitors display temporally regulated and distinct biases in the fates of their progeny. Development.

[B32] Marquardt T, Ashery-Padan R, Andrejewski N, Scardigli R, Guillemot F, Gruss P (2001). Pax6 is required for the multipotent state of retinal progenitor cells. Cell.

[B33] Burmeister M, Novak J, Liang MY, Basu S, Ploder L, Hawes NL, Vidgen D, Hoover F, Goldman D, Kalnins VI, Roderick TH, Taylor BA, Hankins MH, McInnes RR (1996). Ocular retardation mouse caused by Chx10 homeobox null allele: impaired retinal progenitor proliferation and bipolar cell differentiation. Nat Genet.

[B34] Sun H, Chang Y, Schweers B, Dyer MA, Zhang X, Hayward SW, Goodrich DW (2006). An E2F Binding-Deficient Rb1 Protein Partially Rescues Developmental Defects Associated with Rb1 Nullizygosity. Mol Cell Biol.

[B35] Spencer C, Pajovic S, Devlin H, Dinh QD, Corson TW, Gallie BL (2005). Distinct patterns of expression of the RB gene family in mouse and human retina. Gene Expr Patterns.

[B36] Aslanian A, Iaquinta PJ, Verona R, Lees JA (2004). Repression of the Arf tumor suppressor by E2F3 is required for normal cell cycle kinetics. Genes Dev.

[B37] Dyer MA, Livesey FJ, Cepko CL, Oliver G (2003). Prox1 function controls progenitor cell proliferation and horizontal cell genesis in the mammalian retina. Nat Genet.

[B38] Furukawa T, Morrow EM, Cepko CL (1997). Crx, a novel otx-like homeobox gene, shows photoreceptor-specific expression and regulates photoreceptor differentiation. Cell.

[B39] Furukawa T, Morrow EM, Li T, Davis FC, Cepko CL (1999). Retinopathy and attenuated circadian entrainment in Crx-deficient mice. Nat Genet.

[B40] Inoue T, Hojo M, Bessho Y, Tano Y, Lee JE, Kageyama R (2002). Math3 and NeuroD regulate amacrine cell fate specification in the retina. Development.

[B41] Hojo M, Ohtsuka T, Hashimoto N, Gradwohl G, Guillemot F, Kageyama R (2000). Glial cell fate specification modulated by the bHLH gene Hes5 in mouse retina. Development.

[B42] Ohtsuka T, Ishibashi M, Gradwohl G, Nakanishi S, Guillemot F, Kageyama R (1999). Hes1 and Hes5 as notch effectors in mammalian neuronal differentiation. Embo J.

[B43] Dyer MA, Cepko CL (2001). The p57(Kip2) cyclin kinase inhibitor is expressed by a restricted set of amacrine cells in the rodent retina [In Process Citation]. J Comp Neurol.

