# Supplementary material for: Compensation by tumor suppressor genes during retinal development in mice and humans
Source: BMC Biol. 2006 May 3;4:14. doi: 10.1186/1741-7007-4-14 (PMC1481602; doi:10.1186/1741-7007-4-14)
Supplement: Additional file 1 — Expression of the Rb, p107 and p130 during the cell cycle in retinal progenitor cells. [file 1741-7007-4-14-S1.DOC]

**Additional File 1. Expression of the Rb, p107 and p130 during the cell cycle in retinal progenitor cells.**

| Stage | Rb Family | Culture time*^a^* (h) | [^3^H]thy^+^/total  (counts,mean%±SD)*^b^* | Imm^+^/total  (counts, mean%±SD) | Imm^+^,[^3^H]thy^+^/[^3^H]thy^+^  (counts, mean%±SD) | Imm^+^,[^3^H]thy^+^/Imm^+^  (counts, mean%±SD)*^c^* |
| --- | --- | --- | --- | --- | --- | --- |
| E14.5 | p107 | 0 | 68/250,63/250  (26±1.4) | 117/250, 136/250  (50.6±5.3) | 45/68, 40/63  (64.7±1.9) | 45/117, 40/136  (33±3.3) |
| E14.5 | p107 | 4 | 65/250, 60/250  (25 ± 1.4) | 113/250, 101/250  (43 ± 3.4) | 40/65, 38/60  (62 ± 1.2) | 40/113, 38/101  (36 ± 1.6) |
| E14.5 | p107 | 8 | 61/250, 56/250  (23 ± 1.4) | 105/250, 118/250  (45 ± 3.6) | 43/61, 42/56  (72 ± 3) | 43/105, 42/118  (38 ± 3.7) |
| E14.5 | p107 | 16 | 68/250, 61/250  (26 ± 2.0) | 130/250, 113/250  (49 ± 4.8) | 45/68, 54/61  (77 ± 15) | 45/130, 54/113  (41 ± 9) |
| E14.5 | p107 | 24 | 73/250, 77/250  (30 ± 1.1) | 150/250, 138/250  (58 ± 3.4) | 35/73, 39/77  (49 ± 1.9) | 35/150, 39/138  (25 ± 3.4) |
| P0 | Rb | 0 | 60/250, 51/250  (22 ± 2.5) | 150/250, 127/250  (55 ± 6.5) | 54/60, 43/51  (87 ± 4) | 54/150, 43/127  (35 ± 1.5) |
| P0 | Rb | 4 | 52/250, 48/250  (20 ± 1.1) | 147/250, 172/250  (64 ± 7) | 46/52, 39/48  (85 ± 5) | 46/147, 39/172  (27 ± 6) |
| P0 | Rb | 8 | 54/250, 58/250  (22 ± 1.1) | 153/250, 187/250  (68 ± 9.6) | 48/54, 37/58  (76 ± 17) | 48/153, 37/187  (25 ± 8.1) |
| P0 | Rb | 16 | 64/250, 46/250  (22 ± 5.0) | 137/250, 152/250  (58 ± 4.2) | 26/54, 28/46  (54 ± 9) | 26/137, 28/152  (19 ± 0.4) |
| P0 | Rb | 24 | 57/250, 62/250  (24 ± 1.4) | 132/250,150/250  (56 ± 5) | 42/57, 48/62  (75 ± 2.6) | 42/132, 48/150  (32 ± 0.1) |
| P2 | p130 | 0 | 27/500, 23/500  (5 ± 0.5) | 2/500, 1/500  (0.3 ± 0.1) | 0/27, 0/23  (0 ± 0) | 0/21, 1/19  (2.6 ± 3.7) |
| P2 | p130 | 4 | 29/500, 29/500  (5.8 ± 0) | 2/500, 2/500  (0.4 ± 0) | 0/29, 0/29  (0 ± 0) | 0/14, 2/11  (9 ± 12) |
| P2 | p130 | 8 | 24/500, 32/500  (5.6 ± 1.1) | 2/500, 2/500  (0.4 ± 0) | 2/24, 2/32  (7.2 ± 1.4) | 18/20, 14/16  (89 ± 1.7) |
| P2 | p130 | 16 | 37/500, 39/500  (7.6 ± 0.2) | 7/500, 8/500  (1.5 ± 0.14) | 3/37, 3/39  (8 ± 0.3) | 5/15, 6/11  (44 ± 15) |
| P2 | p130 | 24 | 39/500, 42/500  (8.1 ± 0.4) | 11/500, 14/500  (2.5 ± 0.4) | 1/39, 1/42  (2.4 ± 0.1) | 1/13, 2/21  (8.6 ± 1.3) |
|  |  |  |  |  |  |  |
|  |  |  |  |  |  |  |

Shaded column indicates the data used to determine the proportion of proliferating cells expressing p107, Rb or p130. For example, at E14.5 the proportion of retinal progenitor cells expressing p107 ranges from 62-77%.

*^a^* Freshly dissected retinae (three to six) were incubated with [^3^H]thymidine in culture medium for 1 hr, washed, and cultured as explants for the amount of time indicated.

*^b^* The number of grains for 10 randomly selected [^3^H]thymidine-labeled cells varied from 10-19 grains per cell (mean = 15±10). The number of grains for 10 randomly selected unlabeled cells varied from zero to 2.

*^c^* The samples immunostained for Rb and p107 are presented as Imm^+^,[^3^H]thy^+^/Imm^+^. For p130, the proportion of immunopositive cells was too low to use this method to obtain significant data. Instead, for p130 the fields of cells were scanned for p130 immunopositive cells and then the number of silver grains were scored.
