# Supplementary figures and images for: Compensation by tumor suppressor genes during retinal development in mice and humans
Source: BMC Biol. 2006 May 3;4:14. doi: 10.1186/1741-7007-4-14 (PMC1481602; doi:10.1186/1741-7007-4-14)

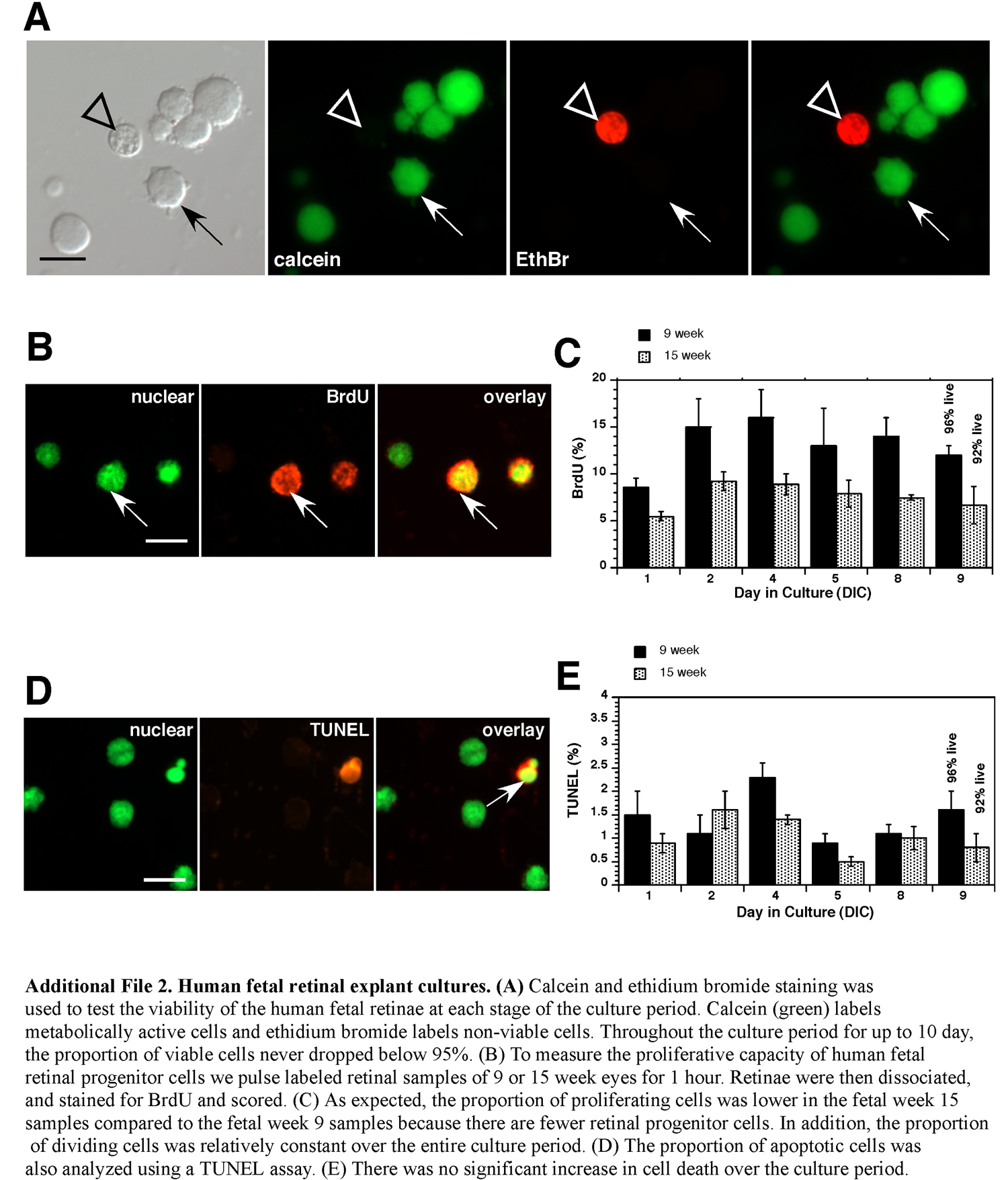

Supplement: Additional file 2 — Human fetal retinal explant cultures. [file 1741-7007-4-14-S2.TIFF]
