# Supplementary material for: Compensation by tumor suppressor genes during retinal development in mice and humans
Source: BMC Biol. 2006 May 3;4:14. doi: 10.1186/1741-7007-4-14 (PMC1481602; doi:10.1186/1741-7007-4-14)
Supplement: Additional file 3 — Effects of acute inactivation of Rb in the developing retina. [file 1741-7007-4-14-S3.DOC]

**Additional File 3. Effects of acute inactivation of *Rb* in the developing retina.**

| **Cell population^a^** | **Plasmid** | **BrdU^+^/total**  **(counts, mean%±SD)** | **[^3^H]-thy^+^/total**  **(counts, mean%±SD)** | **BrdU^+^,[^3^H]-thy^+^/^3^H]-thy^+^**  **(counts, mean%±SD)** |
| --- | --- | --- | --- | --- |
| unsorted | venus | 13/250, 14/250  (5.4±0.1) | 6/250, 8/250  (2.8±0.8) | 1/25, 0/25  (2.0±1) |
| YFP+ | venus | n.d. | 7/500, 6/500  (1.3±0.4) | 0/25,1/25  (2.0±1) |
| YFP– | venus | n.d. | 4/500,2/500  (0.5±0.08) | 1/25,0/25  (2.0±1) |
| unsorted | venus-Cre | 16/250, 14/250  (6.1±0.9) | 5/250, 4/250  (1.8±0.07) | 9/50, 11/50  (21±1.4) |
| YFP+ | venus-Cre | n.d. | 17/500,16/500  (3.2±0.09) | 14/50, 11/50  (24±5) |
| YFP– | venus-Cre | n.d. | 1/500, 1/500  (0.2±0) | 1/25, 0/25  (2.0±1) |

^a^ Total of 250 unsorted cells were scored, and 500 YFP+ or YFP- cells were scored.
