# Supplementary material for: Compensation by tumor suppressor genes during retinal development in mice and humans
Source: BMC Biol. 2006 May 3;4:14. doi: 10.1186/1741-7007-4-14 (PMC1481602; doi:10.1186/1741-7007-4-14)
Supplement: Additional file 4 — Acute inactivation of p107 in the Rb-deficient retinae during development. [file 1741-7007-4-14-S4.DOC]

**Additional File 4. Acute inactivation of *p107* in the *Rb*-deficient retinae during development.**

| Genotype | Plasmid | Cell  Population^a^ | [^3^H]-thy^+^/total | **BrdU^+^/total**  Imm^+^,[^3^H]-thy^+^/[^3^H]-thy^+^  (counts, mean%±SD) | **Chx10^+^/total**  Imm^+^,[^3^H]-thy^+^/[^3^H]-thy^+^  (counts, mean%±SD) | **Pax6^+^/total**  Imm^+^,[^3^H]-thy^+^/[^3^H]-thy^+^  (counts, mean%±SD) | **Syn^+^/total**  Imm^+^,[^3^H]-thy^+^/[^3^H]-thy^+^  (counts, mean%±SD) | **PKC-α+/total**  Imm^+^,[^3^H]-thy^+^/[^3^H]-thy^+^  (counts, mean%±SD) |  |
| --- | --- | --- | --- | --- | --- | --- | --- | --- | --- |
| *Rb^Lox/–^* | P-sil. | YFP^+^ | 3/250, 0/250 | **8/250, 6/250**  n/a | **13/250, 15/250**  n/a | **7/250, 6/250**  n/a | **10/250, 11/250**  n/a | **12/250, 11/250**  n/a |  |
| *Rb^Lox/–^* | P-sil. | YFP^–^ | 0/250, 1/250 | **3/250, 6/250**  n/a | **12/250, 11/250**  n/a | **11/250, 9/250**  n/a | **6/250, 8/250**  n/a | **14/250, 12/250**  n/a |  |
| *Rb^Lox/–^* | P-sil.^p107^ | YFP^+^ | 2/250, 2/250 | **9/250, 10/250**  n/a | **12/250, 12/250**  n/a | **12/250, 12/250**  n/a | **10/250, 9/250**  n/a | **22/250, 16/250**  n/a |  |
| *Rb^Lox/–^* | P-sil.^p107^ | YFP^–^ | 2/250, 0/250 | **4/250, 7/250**  n/a | **11/250, 9/250**  n/a | **8/250, 13/250**  n/a | **10/250, 11/250**  n/a | **9/250, 9/250**  n/a |  |
| *Chx10-Cre;Rb^Lox/–^* | P-sil. | YFP^+^ | 50/250, 43/250 | **8/250, 9/250**  4/25, 5/25  18%±2.8% | **12/250, 13/250**  6/25, 4/25  20%±5.6% | **25/250, 18/250**  7/25, 8/25  30%±2.8% | **27/250, 25/250**  5/25, 8/25  26%±8.4% | **15/250, 17/250**  1/25, 0/25  2%±2.8% |  |
| *Chx10-Cre;Rb^Lox/–^* | P-sil. | YFP^–^ | 41/250, 44/250 | **7/250, 6/250**  3/25, 5/25  16%±5.6% | **14/250, 17/250**  2/25, 5/25  14%±8.4% | **19/250, 20/250**  7/25, 4/25  22%±8.4% | **17/250, 21/250**  7/25, 5/25  24%±5.6% | **18/250, 16/250**  0/25, 2/25  4%±5.6% |  |
| *Chx10-Cre;Rb^Lox/–^* | P-sil.^p107^ | YFP^+^ | 89/250, 82/250 | **25/250, 29/250**  10/25, 9/25  38%±2.8% | **20/250, 24/250**  15/25, 14/25  58%±2.8% | **39/250, 42/250**  12/25, 12/25  48%±0% | **43/250, 49/250**  14/25, 11/25  50%±8.4% | **10/250, 17/250**  2/25, 1/25  6%±2.8% |  |
| *Chx10-Cre;Rb^Lox/–^* | P-sil.^p107^ | YFP^–^ | 37/250, 46/250 | **13/250, 17/250**  5/25, 2/25  14%±8.4% | **12/250, 15/250**  7/25, 6/25  26%±2.8% | **15/250, 13/250**  5/25, 6/25  22%±2.8% | **16/250, 15/250**  3/25, 7/25  20%±11% | **7/250, 11/250**  3/25, 1/25  8%±5.6% |  |
|  |  |  |  |  |  |  |  |  |  |

^a^ A total of 250 cells were scored for each population.

n/a There were too few [^3^H]-thy^+^ cells to score enough cells to report.

Boxed cells indicate ectopic proliferation following acute *p107* gene inactivation.
