# Supplementary material for: Compensation by tumor suppressor genes during retinal development in mice and humans
Source: BMC Biol. 2006 May 3;4:14. doi: 10.1186/1741-7007-4-14 (PMC1481602; doi:10.1186/1741-7007-4-14)
Supplement: Additional file 5 — Expression of Proliferation and Differentiation Markers in P0 Retinae Lacking Rb and/or p107. [file 1741-7007-4-14-S5.DOC]

**Additional File 5. Expression of Proliferation and Differentiation Markers in P0 Retinae Lacking Rb and/or p107.**

| **Ab** | **Control^a^**  **Imm^+^/total**  **(counts, mean%±SD)** | **Control**  **Imm^+^,[^3^H]thy^+^/Imm^+^**  **(counts, mean%±SD)** | ***Rb^–/–^;p107^+/–^* ^b^**  **Imm^+^/total**  **(counts, mean%±SD)** | ***Rb^–/–^;p107^+/–^***  **Imm^+^,[^3^H]thy^+^/Imm^+^**  **(counts, mean%±SD)** | ***Rb^–/–^;p107^–/–^* ^c^**  **Imm^+^/total**  **(counts, mean%±SD)** | ***Rb^–/–^;p107^–/–^***  **Imm^+^,[^3^H]thy^+^/Imm^+^**  **(counts, mean%±SD)** |
| --- | --- | --- | --- | --- | --- | --- |
| BrdU | 22/250, 21/250  (8.6±0.3) | 21/22, 20/21  (95±0.1) | 56/250, 51/250  (21±1.4) | 52/56, 48/51  (93±0.8) | 90/250, 88/250  (36±0.5) | 90/90, 84/88  (98±3.2) |
| Syn | 22/250, 23/250  (9.0±0.3) | 0/150, 0/150  0 | 33/250, 37,250  (14±1.1) | 1/150, 2/150  (1.0±0.4) | 41/250, 31/250  (14±2.8) | 1/150, 1/150  (0.6±0) |
| Pax6 | 16/250, 11/250  (5.4±1.4) | 4/100, 3/100^d^  (3.5±0.7) | 32/250, 38/250  (14±1.6) | 7/100, 6/100  (6.5±0.7) | 45/250, 41/250  (17.2±1.1) | 7/100, 8/100  (7.5±0.7) |
| Rec | 13/250, 19/250  (6.4±1.7) | 0/100, 0/100  0 | 10/250, 7/250  (3.4±0.8) | 0/25, 0/25^e^  0 | 11/250, 6/250  (3.4±1.4) | 0/25, 0/25^5^  0 |
| Cone | 4/250, 6/250  (2.0±0.5) | 0/25, 0/25  0 | 7/250, 4/250  (2.2±0.8) | 0/25, 0/25  0 | 3/250, 3/250  (1.2±0) | 0/25, 0/25  0 |
| p27 | 168/250, 134/250  (60±9.6) | n.d. | 113/250, 115,250  (45±0.5) | n.d. | 118/250, 110, 250  (46±2.2) | n.d. |
| Calb | 1/500, 2/500  (0.3±0.1) | 0/10, 0/10  0 | 1/500, 1/500  (0.2±0) | 0/10, 0/10  0 | 2/500, 2/500  (0.4±0) | 0/10, 0/10  0 |
| Rho | 29/250, 25/250  (10.8±1.1) | 0/100, 0/100  0 | 2/250, 4/250  (1.2±0.5) | 0/25, 0/25  0 | 4/250, 3/250  (1.4±0.3) | 0/25, 0/25  0 |
|  |  |  |  |  |  |  |
|  |  |  |  |  |  |  |
|  |  |  |  |  |  |  |
|  |  |  |  |  |  |  |

^a^ For these analyses, the control was a p107^+/–^ littermate.

^b^ *Rb^–/–^;p107^+/–^* is generated using the lox allele of *Rb* and the *Chx10-Cre* transgene (*Chx10-Cre;Rb^Lox/–^;p107^+/–^*).

^c^ *Rb^–/–^;p107^–/–^* is generated using the lox allele of *Rb* and the *Chx10-Cre* transgene (*Chx10-Cre;Rb^Lox/–^;p107^–/–^*).

^d^ The pax6^+^ cells that also colocalized with [^3^H]-thymidine were fainter than those that did not colocalize with [^3^H]-thymidine.

^e^ In cases where there were not enough immunopositive cells to score 150 cells in the analysis of Imm^+^,[^3^H]thy^+^/Imm^+^ cells, the maximum number of cells that could be scored was used.

Abbreviations: Calb, Calbindin; Rho, Rhodopsin; Rec, Recoverin; Syn, Syntaxin.
