# Supplementary material for: Compensation by tumor suppressor genes during retinal development in mice and humans
Source: BMC Biol. 2006 May 3;4:14. doi: 10.1186/1741-7007-4-14 (PMC1481602; doi:10.1186/1741-7007-4-14)
Supplement: Additional file 6 — Expression of Proliferation and Differentiation Markers in P6 Retinae Lacking Rb and/or p107. [file 1741-7007-4-14-S6.DOC]

**Additional File 6. Expression of Proliferation and Differentiation Markers in P6 Retinae Lacking Rb and/or p107.**

| **Ab** | **Control^a^**  **Imm^+^/total**  **(counts, mean%±SD)** | **Control**  **Imm^+^,[^3^H]thy^+^/Imm^+^**  **(counts)** | ***Rb^+/–^;p107^–/–^***  **Imm^+^/total**  **(counts, mean%±SD)** | ***Rb^+/–^;p107^–/–^***  **Imm^+^,[^3^H]thy^+^/Imm^+^**  **(counts)** | ***Rb^–/–^;p107^+/–^* ^b^**  **Imm^+^/total**  **(counts, mean%±SD)** | ***Rb^–/–^;p107^+/–^***  **Imm^+^,[^3^H]thy^+^/Imm^+^**  **(counts, mean%±SD)** | ***Rb^–/–^;p107^–/–^* ^c^**  **Imm^+^/total**  **(counts, mean%±SD)** | ***Rb^–/–^;p107^–/–^***  **Imm^+^,[^3^H]thy^+^/Imm^+^**  **(counts, mean%±SD)** |
| --- | --- | --- | --- | --- | --- | --- | --- | --- |
| BrdU | 1/250, 0/250  (0.3±0.3) | 1/1, 0/0 | 1/250, 2/250  (0.6±0.3) | 1/1, 2/2 | 11/250, 13/250  (4.8±0.5) | 11/11, 12/13  (96±5.4) | 14/250, 11/250  (5.0±0.8) | 12/14, 11/11  (93±10) |
| PKC | 9/250, 14/250  (4.6±1.4) | 0/100, 0/100 | 10/250, 12/250  (4.4±0.5) | 0/100, 0/100 | 4/250, 3/250  (1.4±0.3) | 0/50, 0/50  0 | 4/250, 6/250  (2.0±0.5) | 0/50, 0/50  0 |
| Pax6 | 14/250, 22/250  (7.2±0.2) | 0/100, 0/100^d^ | 19/250, 26/250  (9±1.9) | 0/100, 0/100 | 69/250, 55/250  (24.8±3.9) | 10/100, 11/100  (10.5±0.7) | 28/250, 41/250  (13.8±3.6) | 12/100, 8/100  (10±2.8) |
| Chx10 | 18/250, 14/250  (6.4±1.1) | 0/100, 0/100 | 28/250, 29/250  (11±0.3) | 0/100, 0/100 | 15/250, 22/250  (7.4±1.9) | 7/100, 8/100  (7.5±0.7) | 27/250, 21/250  (9.6±1.6) | 28/100, 35/100  (31.5±4.9) |
| Tunel | 12/250, 6/250  (3.6±1.7) | n.d. | 5/250, 4/250  (1.8±0.3) | n.d. | 5/250, 6/250  (2.2±0.2) | n.d. | 8/250, 10/250  (3.6±0.5) | n.d. |
| Calb | 1/500, 1/500  (0.2±0) | 0/10, 0/10^e^ | 0/500, 1/500  (0.1±0.1) | 0/10, 0/10^e^ | 1/500, 2/500  (0.3±0.1) | 0/10, 0/10^e^  0 | 1/500, 0/500  (0.1±0.1) | 0/10, 0/10^e^  0 |
| Rho | 134/250, 110/250  (49±6.7) | 0/100, 0/100 | 83/250, 78/250  (32±1.4) | 0/100, 0/100 | 12/250, 15/250  (5.4±0.8) | 0/50, 0/50^e^  0 | 11/250, 14/250  (5.0±0.8) | 0/50, 0/50  0 |
|  |  |  |  |  |  |  |  |  |
|  |  |  |  |  |  |  |  |  |
|  |  |  |  |  |  |  |  |  |
|  |  |  |  |  |  |  |  |  |

^a^ For these analyses, the control was a p107^+/–^ littermate.

^b^ *Rb^–/–^;p107^+/–^* is generated using the lox allele of *Rb* and the *Chx10-Cre* transgene (*Chx10-Cre;Rb^Lox/–^;p107^+/–^*).

^c^ *Rb^–/–^;p107^–/–^* is generated using the lox allele of *Rb* and the *Chx10-Cre* transgene (*Chx10-Cre;Rb^Lox/–^;p107^–/–^*).

^d^ The pax6^+^ cells that also colocalized with [^3^H]-thymidine were fainter than those that did not colocalize with [^3^H]-thymidine.

^e^ In cases where there were not enough immunopositive cells to score 100 cells in the analysis of Imm^+^,[^3^H]thy^+^/Imm^+^ cells, the maximum number of cells that could be scored was used.

Abbreviations: Calb, Calbindin; Rho, Rhodopsin; PKC, protein kinase C.
