# Supplementary material for: Compensation by tumor suppressor genes during retinal development in mice and humans
Source: BMC Biol. 2006 May 3;4:14. doi: 10.1186/1741-7007-4-14 (PMC1481602; doi:10.1186/1741-7007-4-14)
Supplement: Additional file 7 — Expression of Proliferation and Differentiation Markers in P12 Retinae Lacking Rb and/or p107. [file 1741-7007-4-14-S7.DOC]

**Additional File 7. Expression of Proliferation and Differentiation Markers in P12 Retinae Lacking Rb and/or p107.**

| **Ab** | **Control^a^**  **Imm^+^/total**  **(counts, mean%±SD)** | **Control**  **Imm^+^,[^3^H]thy^+^/Imm^+^**  **(counts)** | ***Rb^+/–^;p107^–/–^***  **Imm^+^/total**  **(counts, mean%±SD)** | ***Rb^+/–^;p107^–/–^***  **Imm^+^,[^3^H]thy^+^/Imm^+^**  **(counts)** | ***Rb^–/–^;p107^+/–^* ^b^**  **Imm^+^/total**  **(counts, mean%±SD)** | ***Rb^–/–^;p107^+/–^***  **Imm^+^,[^3^H]thy^+^/Imm^+^**  **(counts, mean%±SD)** | ***Rb^–/–^;p107^–/–^* ^c^**  **Imm^+^/total**  **(counts, mean%±SD)** | ***Rb^–/–^;p107^–/–^***  **Imm^+^,[^3^H]thy^+^/Imm^+^**  **(counts, mean%±SD)** |
| --- | --- | --- | --- | --- | --- | --- | --- | --- |
| BrdU | 0/250, 0/250  0 | 0/0 | 0/250, 0/250  0 | 0/0 | 15/250, 19/250  (6.8±1.1) | 15/15, 18/19  (97±3.7) | 17/250, 15/250  (6.4±0.5) | 16/17, 12/15  (87±9.9) |
| PKC | 25/250, 18/250  (8.6±1.9) | 0/100, 0/100 | 18/250, 17/250  (7±0.2) | 0/100, 0/100 | 31/250, 23/250  (10.8±2.2) | 0/100, 0/100  0 | 14/250, 15/250  (5.8±0.2) | 0/100, 0/100  0 |
| Pax6 | 12/250, 16/250  (8.6±1.9) | 0/100, 0/100 | 13/250, 17/250  (6.0±1.1) | 0/100, 0/100 | 26/250, 22/250  (9.6±1.1) | 6/100, 7/100^d^  (6.5±0.7) | 60/250, 42/250  (20.4±5) | 11/100, 13/100  (12±1) |
| Chx10 | 23/250, 24/250  (9.4±0.2) | 0/100, 0/100 | 18/250, 22/250  (8.01.1) | 0/100, 0/100 | 11/250, 15/250  (5.2±1.1) | 7/50, 6/50  (13±1) | 8/250, 11/250  (3.8±0.8) | 5/50, 6/50  (11±1) |
| GFAP | 0/250, 0/250  0 | n.d. | 0/250, 0/250  0 | n.d. | 3/250, 4/250  (1.4±0.2) | 1/20, 0/20  (2.5±3.5) | 5/250, 7/250  (2.4±0.5) | 2/20, 1/20  (7.5±3.5) |
| Calb | 1/500, 0/500  (0.1±0.1) | 0/10, 0/10^e^ | 0/500, 1/500  (0.1±0.1) | 0/10, 0/10^e^ | 1/500, 1/500  (0.2±0) | 0/10, 0/10^e^  0 | 1/500, 1/500  (0.2±0) | 1/10, 0/10^e^  (5±5) |
| Rec | 152/250, 146/250  (59.6±1.7) | 0/100, 0/100 | 125/250, 136/250  (52±3.1) | 0/100, 0/100 | 38/250, 31/250  (13.8±2.0) | 0/100, 0/100  0 | 20/250, 23/250  (8.6±0.8) | 0/100, 0/100  0 |
| Syn | 19/250, 18/250  (7.4±0.2) | 0/100, 0/100 | 14/250, 18/250  (6.4±1.1) | 0/100, 0/100 | 17/250, 19/250  (7.2±0.5) | 1/100, 2/100^e^  (1.5±0.7) | 36/250, 44/250  (16±2.2) | 10/100, 13/100  (11.5±2.1) |
| Cone | 5/250, 6/250  (2.2±0.2) | 0/50, 0/50 | 6/250, 6/250  (2.4±0) | 0/50, 0/50 | 8/250, 7/250  (3.0±0.2) | 0/50, 0/50  0 | 7/250, 6/250  (2.6±0.2) | 0/50, 0/50  0 |
| GS | 9/250, 10/250  (3.8±0.2) | 0/50, 0/50 | 7/250, 6/250  (2.6±0.2) | 0/50, 0/50 | 8/250, 6/250  (2.8±0.5) | 2/50, 1/50  (3±1) | 8/250, 11/250  (3.8±0.8) | 3/50, 4/50  (7±1.4) |
|  |  |  |  |  |  |  |  |  |
|  |  |  |  |  |  |  |  |  |
|  |  |  |  |  |  |  |  |  |
|  |  |  |  |  |  |  |  |  |

^1^ For these analyses, the control was an Rb^+/–^;p107^+/–^ littermate.

^2^ *Rb^–/–^;p107^+/–^* is generated using the lox allele of *Rb* and the *Chx10-Cre* transgene (*Chx10-Cre;Rb^Lox/–^;p107^+/–^*).

^3^ *Rb^–/–^;p107^–/–^* is generated using the lox allele of *Rb* and the *Chx10-Cre* transgene (*Chx10-Cre;Rb^Lox/–^;p107^–/–^*).

^4^ The pax6^+^ cells that also colocalized with [^3^H]-thymidine were fainter than those that did not colocalize with [^3^H]-thymidine.

^5^ In cases where there were not enough immunopositive cells to score 100 cells in the analysis of Imm^+^,[^3^H]thy^+^/Imm^+^ cells, the maximum number of cells that could be scored was used.

Abbreviations: Calb, Calbindin; PKC, protein kinase C; Rec, Recoverin; Syn, Syntaxin, GS, glutamine synthetase; Cone, Cone Arrestin.
