# Supplementary material for: Compensation by tumor suppressor genes during retinal development in mice and humans
Source: BMC Biol. 2006 May 3;4:14. doi: 10.1186/1741-7007-4-14 (PMC1481602; doi:10.1186/1741-7007-4-14)
Supplement: Additional file 8 — Expression of Proliferation and Differentiation Markers in P30 Retinae Lacking Rb and/or p107. [file 1741-7007-4-14-S8.DOC]

**Additional File 8. Expression of Proliferation and Differentiation Markers in P30 Retinae Lacking Rb and/or p107.**

| **Ab** | **Control^a^**  **Imm^+^/total**  **(counts, mean%±SD)** | **Control**  **Imm^+^,[^3^H]thy^+^/Imm^+^**  **(counts)** | ***Rb^–/–^;p107^+/–^* ^b^**  **Imm^+^/total**  **(counts, mean%±SD)** | ***Rb^–/–^;p107^+/–^***  **Imm^+^,[^3^H]thy^+^/Imm^+^**  **(counts)** | ***Rb^–/–^;p107^–/–^* ^c^**  **Imm^+^/total**  **(counts, mean%±SD)** | ***Rb^–/–^;p107^–/–^***  **Imm^+^,[^3^H]thy^+^/Imm^+^**  **(counts, mean%±SD)** |
| --- | --- | --- | --- | --- | --- | --- |
| BrdU | 0/250, 0/250  0 | 0/0 | 0/250, 0/250  0 | 0/0 | 25/250, 19/250  (8.8±1.6) | 25/25, 18/19  (97±3.7) |
| PKC | 26/250, 22/250  (9.6±1.1) | 0/100, 0/100 | 23/250, 21/250  (8.8±0.5) | 0/100, 0/100 | 23/250, 19/250  (8.4±1.1) | 2/100, 5/100  (3.5±2.1) |
| Pax6 | 15/250, 16/250  (6.2±0.2) | 0/50, 0/50 | 22/250, 23/250  (9.0±0.3) | 0/50, 0/50 | 13/250, 19/250  (6.4±1.6) | 1/50, 1/50^d^  (2±0) |
| Chx10 | 14/250, 16/250  (6.0±0.5) | 0/50, 0/50 | 10/250, 12/250  (4.4±0.5) | 0/50, 0/50 | 16/250, 19/250  (7±0.8) | 0/50, 0/50  0 |
| GFAP | 0/250, 0/250  0 | n.d. | 8/250, 7/250  (3±0.3) | 0/20, 0/20 | 11/250, 14/250  (5±0.8) | 1/20, 1/20  (5±0) |
| Calb | 1/500, 1/500  (0.2±0) | 0/10, 0/10^e^ | 2/500, 1/500  (0.3±0.1) | 0/10, 0/10^e^ | 0/500, 1/500  (0.1±0.1) | 1/10, 0/10^e^  (5±7) |
| Rec | 157/250, 164/250  (27±1.9) | 0/100, 0/100 | 41/250, 40/250  (16.2±0.2) | 0/100, 0/100 | 50/250, 46/250  (19.2±1.1) | 5/100, 3/100  (4±1.4) |
| Syn | 22/250, 19/250  (8.2±0.8) | 0/50, 0/50 | 16/250, 20/250  (7.2±1.1) | 0/50, 0/50^e^ | 31/250, 36/250  (13.4±1.4) | 11/50, 12/50  (23±1.4) |
| Cone | 4/250, 3/250  (1.4±0.2) | 0/20, 0/20 | 6/250, 3/250  (1.8±0.8) | 0/20, 0/20 | 6/250, 5/250  (2.2±0.3) | 0/20, 0/20  0 |
| GS | 7/250, 11/250  (3.6±1.1) | 0/20, 0/20 | 5/250, 7/250  (2.4±0.5) | 0/20, 1/20 | 10/250, 9/250  (3.8±0.3) | 1/20, 0/20  (2.5±3.5) |
|  |  |  |  |  |  |  |
|  |  |  |  |  |  |  |
|  |  |  |  |  |  |  |
|  |  |  |  |  |  |  |

^a^ For these analyses, the control was a p107^+/–^ littermate.

^b^ *Rb^–/–^;p107^+/–^* is generated using the lox allele of *Rb* and the *Chx10-Cre* transgene (*Chx10-Cre;Rb^Lox/–^;p107^+/–^*).

^c^ *Rb^–/–^;p107^–/–^* is generated using the lox allele of *Rb* and the *Chx10-Cre* transgene (*Chx10-Cre;Rb^Lox/–^;p107^–/–^*).

^d^ The pax6^+^ cells that also colocalized with [^3^H]-thymidine were fainter than those that did not colocalize with [^3^H]-thymidine.

^e^ In cases where there were not enough immunopositive cells to score 100 cells in the analysis of Imm^+^,[^3^H]thy^+^/Imm^+^ cells, the maximum number of cells that could be scored was used.

Abbreviations: Calb, Calbindin; PKC, protein kinase C; Rec, Recoverin; Syn, Syntaxin, GS, glutamine synthetase; Cone, Cone Arrestin.
