# Supplementary material for: Compensation by tumor suppressor genes during retinal development in mice and humans
Source: BMC Biol. 2006 May 3;4:14. doi: 10.1186/1741-7007-4-14 (PMC1481602; doi:10.1186/1741-7007-4-14)
Supplement: Additional file 9 — Clone composition and distribution from in vivo lineage analysis. [file 1741-7007-4-14-S9.DOC]

**Additional File 9. Clone composition and distribution from in vivo lineage analysis.**

| Virus | Genotype | Rod | **Defective Rod** | Bipolar | **Müller** | **Amacrine** | **^1^Hyperproliferative** |
| --- | --- | --- | --- | --- | --- | --- | --- |
| LIA | *Rb^Lox/–^;p107^–/–^* | 169/231;73% | 0/231;0% | 37/231;16% | 17/231;7% | 8/231;3% | 0/231;0% |
| LIA-Cre | *Rb^Lox/+^;p107^+/–^* | 128/158;81% | 0/158;0% | 11/158;7% | 10/158;6% | 5/158;3% | 0/158;0% |
| LIA-Cre | *Rb^Lox/–^;p107^–/–^* | 67/211;31% | 57/211;27% | 21/211;10% | 9/211;4% | 6/211;3% | 33/211;16% |

^1^ Hyperproliferative lesions were > 50 microns in diameter.
