# Supplementary material for: Compensation by tumor suppressor genes during retinal development in mice and humans
Source: BMC Biol. 2006 May 3;4:14. doi: 10.1186/1741-7007-4-14 (PMC1481602; doi:10.1186/1741-7007-4-14)
Supplement: Additional file 10 — Antibodies used for immunofluorescence on adult tissue. [file 1741-7007-4-14-S10.DOC]

| Antibody | Epitope | Cell Type/Structure | Species^a^ | Dilution^b^ | Source |  |
| --- | --- | --- | --- | --- | --- | --- |
| Rho4D2 | rhodopsin | rod | mouse | 1:500/1:2000 | Molday, R. |  |
| Recoverin | recoverin | rod/cone/rod bipolar | rabbit | 1:200/1:2000 | McGinnis, J.F. |  |
| C10.C10 | rod arrestin | rod | rabbit | 1:100/1:1000 | Craft, C. |  |
| mCAR | cone arrestin | cone | rabbit | 1:200/1:2000 | Craft, C. |  |
| S-opsin | S-opsin | S cone | rabbit | 1:100/1:2000 | Craft, C. |  |
| M-opsin | M-opsin | M cone | rabbit | 1:100/1:2000 | Craft, C. |  |
| CD44 | CD44 | Müller apical microvilli | rat | 1:100/1:1000 | Hybridoma Bank |  |
| Prox1 | Prox1 | Horizontal/AII amacrine | rabbit | 1:100/1:1000 | Oliver, G. |  |
| CL300 | calbindin | Horizontal/subset amacrine | mouse | 1:500/1:5000 | Sigma |  |
| HPC1 | syntaxin | Horizontal/amacrine | mouse | 1:500/1:5000 | Sigma |  |
| NE14 | neurofilament | Horizontal/ganglion processes | mouse | 1:200/1:2000 | Sigma |  |
| VC1.1 | HNK1/NCAM | amacrine processes | mouse | 1:500/1:2000 | Sigma |  |
| Gαo | Gαo | opl | rabbit | 1:100/1:1000 | Santa Cruz |  |
| Bassoon | bassoon | opl/ipl | mouse | 1:400/1:2000 | Stressgen |  |
| SNAP-25 | SNAP-25 | opl | rabbit | 1:100/1:1000 | Stressgen |  |
| K2.4 | Kinesin II | opl | mouse | 1:200/1:2000 | Covance |  |
| 7E3-1B8 | PSD-95 | opl | mouse | 1:100/1:1000 | ABR Inc. |  |
| M4 | PKC-α | bipolar | mouse | 1:100/1:2000 | Upstate |  |
| 115A10 | N/A | bipolar | mouse | 1:1/1:500 | Fujita, S.C. |  |
| Chx10 | Chx10 | bipolar | sheep | 1:100/1:2000 | Exalpha Bio |  |
| PEP19 | Purkinje cell | bipolar/amacrine | rabbit | 1:100/ND | Morgan, J. |  |
| L7 | Purkinje cell | bipolar | rabbit | 1:100 | Morgan, J. |  |
| Glut. Synthetase | Glut. Synthetase | Müller Glia | mouse | 1:100/1:1000 | BD Bioscience |  |
| GA-5 | GFAP | Astrocytes/activated Müller glia | mouse | 1:200/1:1000 | Sigma |  |
| Cyclin D3 | Cyclin D3 | Müller Glia | mouse | 1:100/1:1000 | BD Bioscience |  |
| p27^Kip1^ | p27^Kip1^ | Müller Glia | mouse | 1:20/1:2000 | BD Bioscience |  |
| CD31 | PECAM | Vascular endothelial | mouse | 1:50/ND | Pharmingen |  |
| Dab1 | Disabled 1 | AII amacrine | rabbit | 1:100/ND | Curran, T. |  |
| Calretinin | calretinin | Horizontal/Amacrine subset | mouse | 1:200/1:5000 | Sigma |  |
| ChAT | ChAT | Starburst amacrine | rabbit | 1:50/1:500 | Chemicon |  |
| TH | TH | Dopaminerigic Amacrine | rabbit | 1:100/1:500 | Pel-Freeze |  |
| Six3 | Six3 | Amacrine and bipolar | rabbit | 1:200/1:1000 | Oliver, G. |  |
| Pax6 | Pax6 | Amacrine | mouse | 1:500/1:5000 | Cepko, C. |  |
| Brn 3b | Brain 3b | ganglion | goat | 1:100/1:1000 | Santa Cruz |  |
| Gap7B10 | Gap43 | ganglion | mouse | 1:100/1:1000 | Sigma |  |
| p57^Kip2^ | p57^Kip2^ | Amacrine subset | goat | 1:50/1:1000 | Santa Cruz | |

**Additional File 10** Antibodies used for immunofluorescence on adult tissue.

^a^Organism in which antisera were raised.

^b^ First dilution is for use on tissue sections, second dilution is for use on dissociated retinae and ND indicates the optimal dilution has not yet been determined.
